# Heteroresistance in *Enterobacter cloacae* complex caused by variation in transient gene amplification events

**DOI:** 10.1038/s44259-025-00082-7

**Published:** 2025-02-22

**Authors:** Johannes Kupke, Julian Brombach, Yuwen Fang, Silver A. Wolf, Lakshmipriya Thrukonda, Fereshteh Ghazisaeedi, Benno Kuropka, Dennis Hanke, Torsten Semmler, Niclas Nordholt, Frank Schreiber, Karsten Tedin, Antina Lübke-Becker, Ulrich K. Steiner, Marcus Fulde

**Affiliations:** 1 Department of Veterinary Medicine, Institute of Microbiology and Epizootics, Centre for Infection Medicine, Freie Universität Berlin, Berlin, Germany; 2 Robert Koch Institute (RKI), MF1-Genome Competence Centre, Berlin, Germany; 3 Institute of Chemistry and Biochemistry, Department of Biology, Chemistry, and Pharmacy, Freie Universität Berlin, Berlin, Germany; 4 Federal Institute for Materials Research and Testing (BAM), Department of Materials and the Environment, Division of Biodeterioration and Reference Organisms (4.1), Berlin, Germany; 5 Veterinary Centre for Resistance Research (TZR), Freie Universität Berlin, Berlin, Germany; 6 Institute of Biology, Evolutionary Demography, Freie Universität Berlin, Berlin, Germany

**Keywords:** Antibiotics, Bacteriology

## Abstract

Heteroresistance (HR) in bacteria describes a subpopulational phenomenon of antibiotic resistant cells of a generally susceptible population. Here, we investigated the molecular mechanisms and phenotypic characteristics underlying HR to ceftazidime (CAZ) in a clinical *Enterobacter cloacae* complex strain (ECC). We identified a plasmid-borne gene duplication-amplification (GDA) event of a region harbouring an *ampC* gene encoding a β-lactamase *bla*_DHA-1_ as the key determinant of HR. Individual colonies exhibited variations in the copy number of the genes resulting in resistance level variation which correlated with growth onset (lag times) and growth rates in the presence of CAZ. GDA copy number heterogeneity occurred within single resistant colonies, demonstrating heterogeneity of GDA on the single-cell level. The interdependence between GDA, lag time and antibiotic treatment and the strong plasticity underlying HR underlines the high risk for misdetection of antimicrobial HR and subsequent treatment failure.

## Introduction

Antimicrobial resistance (AMR) poses severe threats to human, animal, and environmental health^1^. In 2019, antibiotic resistant bacteria were associated with the deaths of 5 million people^2^. More frightening, it has been estimated that within the next 30 years this number will increase up to 10 million annual deaths, which would surpass the number of deaths due to prevailing non-communicable diseases, such as cancer and diabetes, respectively^2,3^. In addition to the health consequences, the AMR crisis also poses considerable economic problems for the world. The World Bank estimates that the healthcare costs caused by AMR could exceed 1 trillion US dollars by 2030^4^. It is not without reason that AMR is seen as one of the greatest medical threats to humanity this century.

A major reason for the antibiotic crisis has been the misuse and overuse of antibiotics^5,6^. In order to reduce antimicrobial use as much as feasible, it is essential to reliably and efficiently detect bacterial pathogens and their repertoire of AMR determinants. With no end in sight to the antibiotic crisis and stagnant development of new antibiotics^7^ it is important to understand antibiotic resistance, including resistance phenomena mediated by bacterial subpopulations^8^.

Classical antibiotic resistance is based on a specific resistance factor that leads to stable, heritable resistance. Here, mutations or horizontal transfer of resistance genes, represented in the whole population, confer resistance^9^. However, antibiotic resistance can also be conferred by subpopulations that express different phenotypic traits^10^. One strategy of subpopulations is the formation of persister cells. Bacterial persister cells endure periods of antibiotic treatment by entering a low- or non-growing metabolic state. After withdrawal of the antibiotic, bacteria resume growth but remain susceptible to antibiotic treatments^11^. Another phenomenon is the property of HR, whereby subpopulations of bacteria are able to grow in the presence of an antibiotic while the majority of the susceptible cell population is killed. Importantly, the heteroresistant phenotype can rapidly become a fully resistant population over time under antibiotic selection, leading to treatment failure^8^.

HR was first observed in 1947^12^ and occurs in both Gram-positive and Gram-negative bacteria in response to a broad spectrum of antibiotics^13^. In addition to emerging from a subpopulation, this phenomenon is complex due to the varying antibiotic resistance levels of resistant subpopulations and their ability to revert back to susceptibility. Finally, HR can arise from monoclonal populations, in which heterogeneity arises from genetically identical cells or polyclonal, where a subpopulation of cells harbouring resistance determinants is selected upon antibiotic treatment^14^. Thus, HR poses a challenge to conventional phenotypic AMR testing, where bacteria are generally classified as either resistant or susceptible with respect to the whole population^15^. Phenomena on a subpopulational level are not sufficiently detected by standard, culture-dependent techniques and may require single cell approaches^16^. As a consequence, HR is frequently undetected, leading to misinterpretations regarding the determination of the minimum inhibitory concentration (MIC) used in standard laboratories^17^. HR results in treatment failure, recurrent infections and increased mortality rates of infected individuals^18^.

To reliably detect heteroresistant bacteria, a better understanding of the molecular mechanisms leading to the low-level and transient forms of resistance is required. Of particular interest are bacteria of hospital acquired infections that often show resistance to last resort antibiotics. Among these so-called ESKAPE bacteria is *Enterobacter* spp, with HR to colistin, carbapenems and tigecycline^15,19–21^. HR to colistin has been studied in *Enterobacter* spp. and found to be mediated by the PhoPQ two component system, *ecr* and an inner membrane protein, DedA^22–24^.

HR can be stable or unstable^14^. Unstable gene duplication-amplifications (GDA) have been frequently reported in HR^25^. GDA has previously been shown to confer protection against different stressors such as heat^26^, starvation^27^, heavy metals and pesticides^28^. In bacterial subpopulations, GDA can also lead to antibiotic resistance *via* gene copy number effects for genes encoding antibiotic degrading enzymes^29^, efflux pumps^30^ or other resistance conferring genes, e.g. two component regulatory systems^31^. GDA modulate gene expression by simple copy number accumulation of genes^32^, but also increase the probability of acquiring genetically fixed, point mutations that can provide stable, long term resistance^33,34^. Despite the fact that GDA is known to contribute to bacterial survival under multiple conditions, the underlying mechanisms of GDA and its associated population dynamics in HR remain elusive.

Here, we investigate a veterinary, clinical *Enterobacter cloacae* complex (ECC) strain that was isolated from a horse. The strain exhibits monoclonal HR to the third-generation cephalosporin ceftazidime (CAZ) mediated by GDA of an AmpC β-lactamase encoded by a plasmid-borne copy of *bla*_DHA-1_. We show that GDA of a 17 kilo base pair (kbp) region located on a plasmid is causative for HR against CAZ. Cell-to-cell variations in GDA copy number underpin genomic plasticity that directly translates into phenotypic variation in antibiotic resistance and growth. Furthermore, GDA confers a fitness advantage in the presence of CAZ by shortening the lag-time and increasing growth rates. In the absence of antibiotic selection, the heteroresistant subpopulations harbouring GDAs are outcompeted. We reveal a complex interdependence between fitness, stressor (antibiotic) and GDAs, contributing to the volatile behaviour of resistant subpopulations. Our findings contribute to a better comprehension of the impact of resistant subpopulations on the detection of HR and thus on treatment failure.

## Results

### Identification and characterisation of the heteroresistent Enterobacter cloacae complex (ECC) isolate IMT 49658

**Fig. 1:**
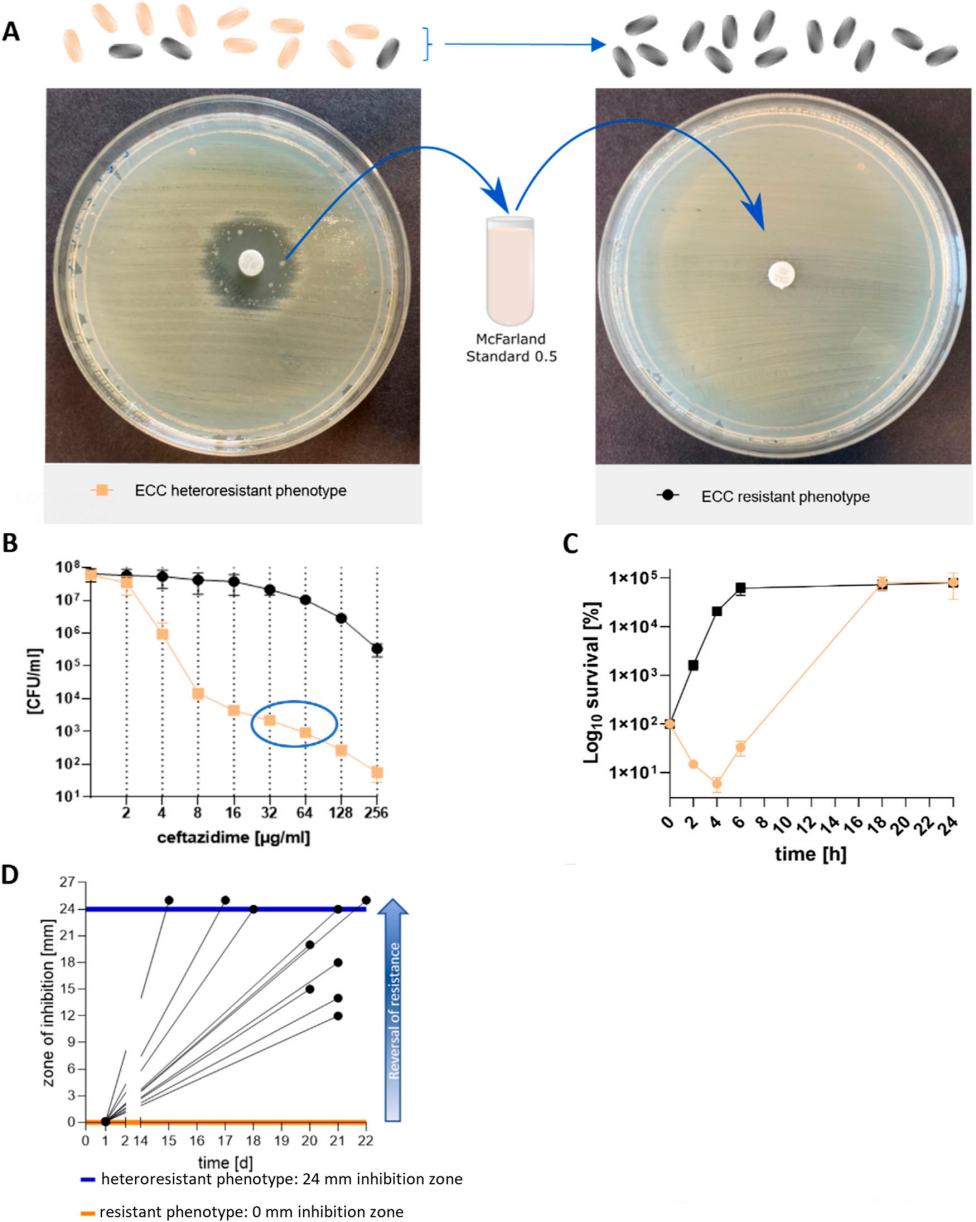
Phenotypic characterisation of heteroresistance (HR) in *Enterobacter cloacae* complex (ECC). **A** In the presence of ceftazidime (CAZ) the source population of ECC exhibits a heteroresistant phenotype (left) in agar-disk diffusion with a susceptible population (outside inhibition zone, beige bacteria) and single colonies within the inhibition zone (black bacteria, resistant subpopulation). Suspending an inhibition zone colony to McFarland standard density and re-plating it results in a resistant phenotype (right). **B** Population analysis profile (PAP) of the source population of ECC (beige) meets the criteria for HR with cell growth on antibiotic plates with 2- or 4 fold breakpoint concentration (blue circle). The size of the resistant subpopulations (y axis, log_10_ scale) on plates with increasing antibiotic concentrations (x axis) is plotted with mean and SD of colony count of three biological replicates. The resistant phenotype preserves its population successfully (black). **C** Time kill assay with log_10_ survival in percent (y-axis) of the resistant (black) and heteroresistant phenotype (beige) over the course of 24 h (x-axis) at 4 × MIC (6 µg/ml CAZ) of the susceptible source population. Values are indicated with mean and SD of three biological replicates. In contrast to the resistant phenotype, the heteroresistant phenotype requires the selection of resistant subpopulations (4–6 h) prior to population growth. **D** Replicates (*n* = 10, black lines) of the resistant phenotype of the *Enterobacter cloacae* complex lose their resistance by subcultivating individual colonies every day (x-axis) on blood plates without CAZ after overnight incubation. The diameter of the inhibition zone (y-axis) of the subcultured replicates was measured using agar-disk diffusion assays. Black dots indicate final measurements of each replicate; horizontal lines indicate the size of the inhibition zone of the ECC source population (blue) and the resistant subpopulation (orange).

During routine diagnostic, we identified a clinical ECC isolate that showed plenty of individual colonies within and distinct from the inhibition zone in an agar-disk diffusion assay with the third-generation cephalosporine CAZ as the antibiotic to be tested. We subsequently purified and re-tested these colonies against CAZ and observed an entirely resistant phenotype (Fig. 1A). The occurrence of resistant individual colonies is a characteristic of HR and provides initial indications of heterogeneity in the overall bacterial population. However, since the detection of HR in agar-disk diffusion assays often yields false-positives^25,35^, we additionally performed time-kill and population analysis profile (PAP) assays^13^. PAP assays confirmed the heteroresistant phenotype of ECC IMT 49658 during CAZ exposure (Fig. 1B) with a subpopulation-size >0.0001% on 2 and 4 times the breakpoint concentration (The clinical breakpoint indicates the antibiotic concentration at which the infection is considered as resistant)^14,36^. In a time-kill assay, the ECC isolate similarly showed a growth pattern characteristic for HR^36,37^. The majority of cells were killed within the first 2 h of treatment, whereas a resistant subpopulation arose between 4 and 24 h and grew to levels comparable to the resistant phenotype (Fig. 1C). HR is further characterised by its reversibility: in the absence of the stressor (in this case, the antibiotic CAZ), the entirely resistant subpopulation reverts back to an almost susceptible population^14^. To determine whether this phenomenon also holds true for ECC IMT 49658, we subcultured ten individual resistant colonies on blood agar plates without antibiotic over the course of 3 weeks. The diameter of the inhibition zone on MH II agar plates were determined in parallel (Supplementary Fig. S1). As depicted in Fig. 1D, all ten resistant replicates revert back to a susceptible phenotype with distinct inhibition zone diameters. Interestingly, all ten replicates showed varying degrees of reversal at the end of the experiment, confirming the heterogenetic nature of this phenomenon (Fig. 1D).

Finally, we asked whether the resistance determinant underlying the resistant phenotype is associated with a fitness loss, as was often observed^38^. Indeed, the resistant phenotype in the ECC isolate appeared to confer a fitness disadvantage in the absence of antibiotics as determined *via* competition assays. Here, the ECC source population outcompeted the resistant phenotype with competition indices of 1.69, 2.03 and 2.64, after 8, 24 and 48 h of competition, respectively (Supplementary Fig. S8).

### Proteomic and genomic analysis reveals high similarities between resistant phenotype and ECC source population

**Fig. 2:**
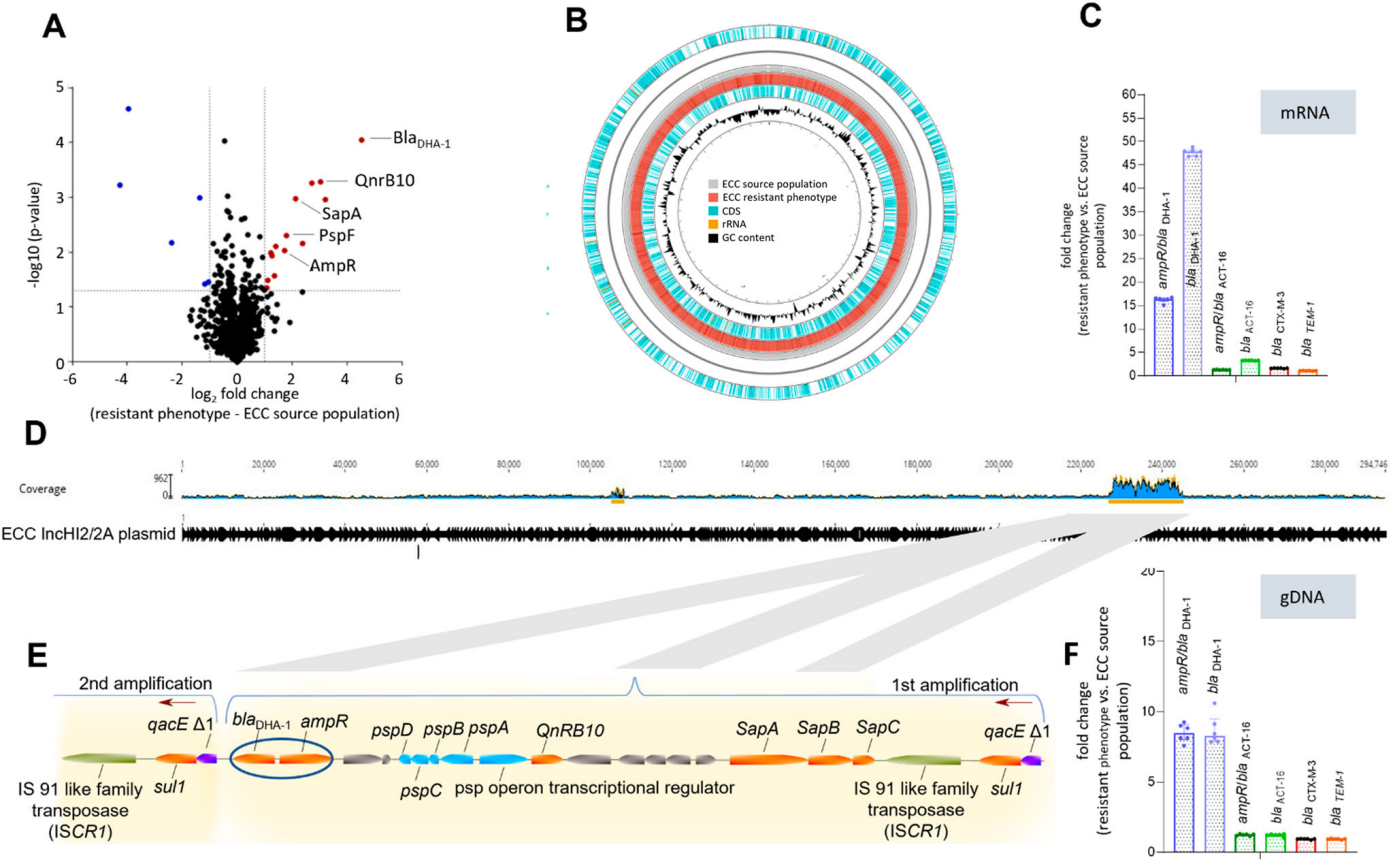
Genetic background of heteroresistance in *Enterobacter cloacae* complex (ECC). **A** Volcano plot of the relative changes in protein signal intensity between the resistant phenotype (right side) and the original ECC source population in the absence of CAZ (left side). Significant protein hits (*p* < 0.05) showing at least a 2-fold change in their relative intensity are marked with blue and red dots. A list of all significant protein hits is shown in Supplementary File 1, Fig. 2. **B** Ring comparison of the complete genomes from resistant phenotype (red) and ECC source population (grey), including coding sequences (CDS, light blue), rRNA (orange) and GC-content (black). Each genomic region of the resistant phenotype has a corresponding region in the ECC source population. **C** Fold change gene expression in the resistant phenotype of 4 different β-lactamases (*bla*_DHA-1_, *bla*_ACT-16_, *bla*_CTX-3_, *bla*_TEM-1_) and two corresponding transcriptional regulators (*ampR/ bla*_DHA-1_, *bla*_ACT-16_) relative to the ECC source population. qPCR results from cDNA were plotted with mean and IQR from *n* = 6 replicates per gene. *Bla*_DHA-1_ and its transcriptional regulator is predominant in the resistant phenotype representing inhibition zone colonies. **D** Mapping of Illumina reads to the reference sequence of plasmid IncHI2/2A reveals a high coverage (blue) for ~17 kbp gene region around position 220.000. **E** The 17 kbp long fragment contains *ampR* and *bla*_DHA-1_ (blue circle) and is amplified in a concatenate manner, confirmed in Oxford MinIon nanopore long read sequencing. The red arrows represent the start of a new gene duplication-amplification (GDA). **F** Fold change copy number in the resistant phenotype of 4 different β-lactamases and two corresponding transcriptional regulators relative to the ECC source population shows a higher copy number for genes from the GDA (*bla*_DHA-1_ and its *ampR*), acquired from qPCR with genomic (g)DNA. Median and IQR of *n* = 6 replicates per gene is plotted.

To clarify the mechanisms underlying the heteroresistant phenotype, we performed quantitative proteome analysis of both an isolate of the resistant phenotype (originating from the CAZ zone of inhibition) and its parental strain (hereafter referred to as ECC susceptible source population) using liquid chromatography-mass spectrometry (LC-MS). Compared to the ECC source population, six proteins were down- and fourteen were up-regulated in the resistant phenotype (Fig. 2A, List of proteins added in Supplementary Fig. S2). Intriguingly, the AmpC-type β-lactamase *bla*_DHA-1_ gene product and *ampC* transcriptional regulator AmpR were both up regulated in the resistant phenotype. This is consistent with the observations that avibactam suppresses the occurrence of inhibition zone colonies in an Epsillon (E-test): Avibactam is a broad spectrum, non-β-lactam inhibitor of both class A- and C-type β-lactamase and has been shown to increase the efficacy of CAZ in over-producing isolates and at high bacterial inocula^39^ (Supplementary Fig. S3).

We next performed a genomic analysis of both the resistant phenotype and the ECC source population after Illumina and Oxford Nanopore sequencing. In both, the resistant phenotype and ECC source population, whole-genome sequencing (WGS) confirmed the presence of class A β-lactamases *bla*_TEM-1_ and *bla*_CTX-M-3_, as well as AmpC β-lactamases, *bla*_ACT-16_ and *bla*_DHA-1._ In addition, Nanopore-sequencing revealed that *bla*_DHA-1_ and the two class A β-lactamases were localised on a ≈290 kbp large IncHI2/2A plasmid, whereas *bla*_ACT-16_ was located on the chromosome. A direct comparison between the ECC source population and the resistant phenotype using the Geneious mauve alignment tool revealed a genetic identity of 99.68% (Fig. 2B). Further, variant calling with Snippy identified no SNPs in genes associated with antibiotic resistance to cephalosporines, such as *ampD* or the promotor regions of AmpC β-lactamases, that might explain the resistant phenotype^40,41^. Both proteomic and genomic data therefore indicated that the HR phenotype was linked to *bla*_DHA-1_ expression but was not due to point mutations in the known resistance genes. Using targeted mutagenesis, we deleted the *bla*_DHA-1_ gene and performed agar-disk diffusion as described above. The isogenic Δ*bla*_DHA-1_-mutant of the ECC source population completely lost its ability to form individual colonies within the inhibition zone and showed no HR on a PAP-Assay according to the definition of Band et al.^36^ (Supplementary Fig. S4B2, B3). The same result occurred in four replicates of ECC IMT 49658 with the deletion of two regions (~51.4 kbp and ~13.6 kbp) of the IncHI/2 A plasmid after prolonged incubation at high temperatures^42^, which led to the elimination of *bla*_DHA-1_ (Supplementary Fig. S4A1, A2)_._ These results finally confirmed that *bla*_DHA-1_ is responsible for the heteroresistent phenotype of ECC IMT 49658.

### AmpC β-lactamase bla_DHA-1_ is amplified within a 17 kbp large gene fragment on the IncHI2/2A plasmid causing HR in ECC

Consistent with the proteomic data, the expression levels of the transcriptional regulator *ampR* and of *bla*_DHA-1_ were 16.37-fold and 47.9-fold higher in the resistant phenotype than in the ECC source population, respectively (Fig. 2C). In contrast, the fold change differences for the three additional β-lactamases, including two ESBL, were negligible. In order to explain the increased expression of *AmpR* and *bla*_DHA-1_, we hypothesised that GDA events might be responsible for the differences in gene expression. Closer inspection of the genome sequencing data from the resistant phenotype showed a higher sequence coverage for a 17 kbp region on the IncHI2/2A plasmid harbouring genes encoding proteins previously identified as up-regulated in the proteomics data, including *bla*_DHA-1_, *qnrB* and the *psp* and *sap* operons (Fig. 2D, E). We confirmed the higher coverage by analysing Nanopore long reads, displaying GDA of the 17 kbp region and using genomic DNA in qPCR. (Fig. 2F). Here, the apparent copy numbers of *ampR* and *bla*_DHA-1_ were between 8.5 and 8.7 times higher in the resistant phenotype than in the ECC source population, respectively. MinION nanopore long read sequencing (Oxford Nanopore Technologies) confirmed that both *bla*_DHA-1_ and its associated *ampR* gene were located on the same GDA region that was flanked by *qacE/sul1* on both sides (Fig. 2E). Additionally, qPCR results of five other genes within the GDA region from independent HR isolates demonstrated similar increased copy numbers within the 17 kbp region (Supplementary Table S3). Since the formation of GDAs can occur in a *recA* dependent or independent manner^43,44^, we subsequently generated a targeted, *recA* deficient ECC mutant strain. When growing a Δ*recA* mutant of the ECC source population overnight in 16 µg/ml CAZ, no GDAs were detected in qPCR, suggesting that the establishment of GDAs in the ECC is a *recA*-dependent mechanism (Supplementary Fig. S4C1, C2).

### Resistant subpopulations exhibit colony—and growth variability linked with the copy number of gene-duplication-amplifications (GDA) that vary on a single cell level

**Fig. 3:**
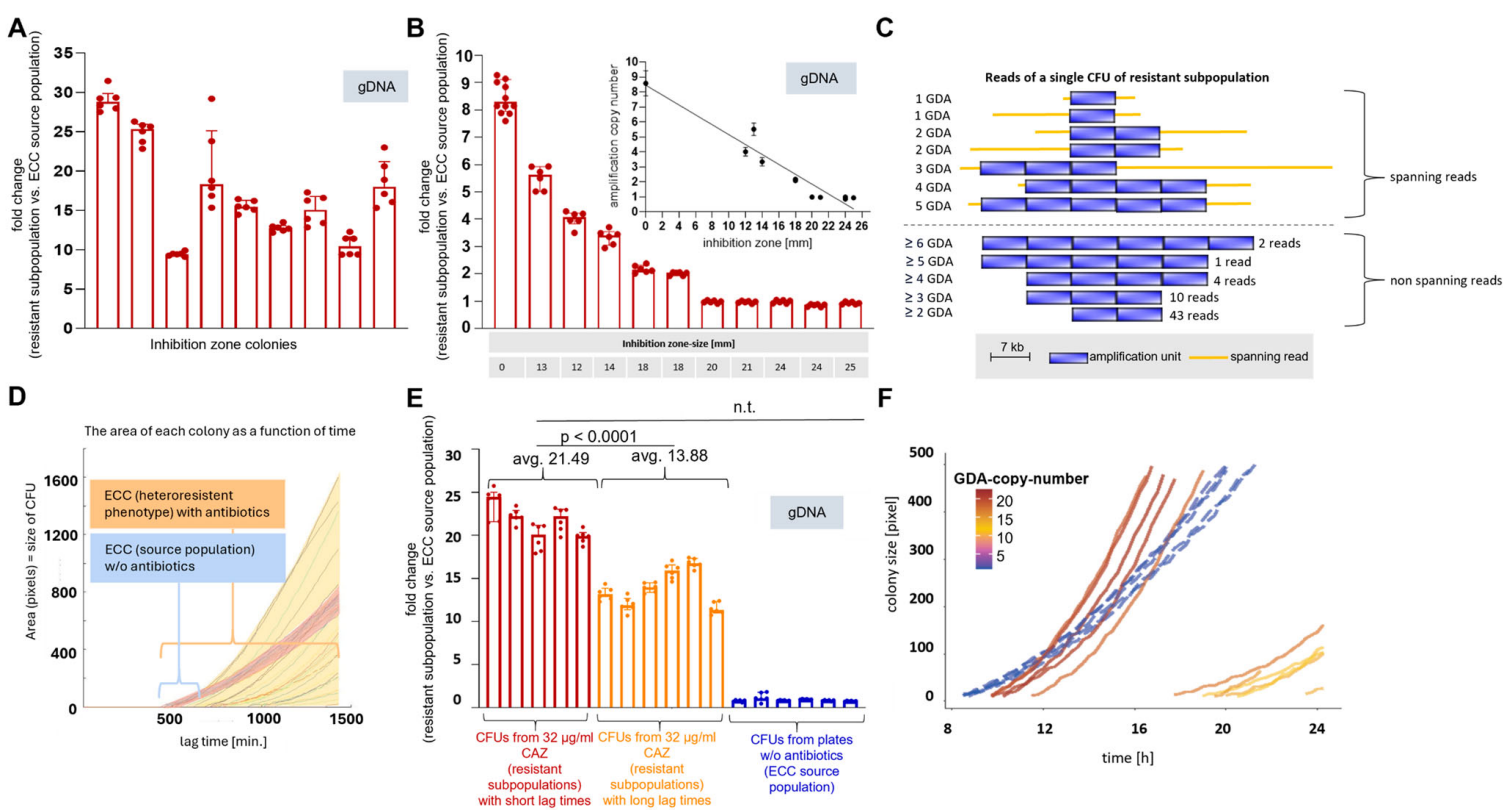
Growth variability of resistant subpopulations via. gene duplication-amplification (GDA) copy number. **A** GDA-copy-number of 9 inhibition zone colonies (IZC) from agar-disk diffusion with CAZ differs between each IZC. qPCR was used to assess GDA copy number with gDNA. The graph displays median (bars) & interquartile ranges (IQR) (error bars) of three technical replicates of *ampR* and *bla*_DAH-1_ per IZC. Dots indicate specific values of technical replicates for *ampR* and *bla*_DHA-1_ genes. 2^-ΔΔct^ values of IZCs represent relative fold change values to cell material outside the inhibition zone. **B** For GDA-copy-number (y-axis) and inhibition zone-size (table at the bottom) of eleven different resistant subpopulations (red bars), a negative correlation (spearman correlation coefficient *r* = −0.94, *p* < 0.0001, CI −0.98 to −0.76) was detected (see inset). **C** GDA-copy-number heterogeneity in reads from Oxford Nanopore MinIon sequencing of a single IZC. Blue bars indicate copies, orange lines represent spanning reads, displaying the entire GDA and genome-areas beyond. Numbers on the right of non-spanning reads indicate the read-amount with the respective GDA-copy-number. Copy-numbers are shown on the left. **D** Area (y-axis) of single colonies (lines) as a function of time (x-axis) for the ECC source population (red shading) without CAZ and with 32 μg/ml CAZ, representing resistant subpopulations (yellow shading) in ScanLag. ECC source population shows homogeneous lag times on plates without antibiotics but heterogeneous lag times when grown on 32 µg/ml CAZ, revealing resistant subpopulations. **E** Fold-change GDA-copy-number from resistant subpopulations relative to the ECC source population, presented by *n* = 5 single colonies in ScanLag emerging early (dark red bars) and *n* = 6 colonies growing late (light red bars) on plates with 32 µg/ml CAZ. CFUs with short lag times have more GDAs than late occurring CFUs. No GDAs occur in CFUs from antibiotic-free plates (blue bars, *n* = 5). **F** CFU size over time, each line represents one CFU. Line colours represent the GDA-copy-number per CFU as defined by the colour scale. Solid lines are resistant CFUs growing on 32 µg/ml CAZ plates in ScanLag. Dashed lines are controls of the source population grown without CAZ.

Next, we speculated about a direct interdependency between gene copy number, expression of resistance and growth behaviour of resistant subpopulations, because we made the observation that independent colonies within the inhibition zone harbour different amounts of GDAs. To address this question, we picked nine independent colonies from within the inhibition zone that occur in variable sizes and at different distances from the CAZ-disk (Fig. 1A, Supplementary Fig. S5). Subsequent qPCR analysis revealed GDA levels ranging from ~9 to ~29 copies (Fig. 3A, Supplementary Fig. S5 and Supplementary Table S4). Moreover, for eleven different resistant subpopulations from the above mentioned reversal experiments, a negative correlation between GDA copy number and the diameter of the inhibition zone was confirmed by qPCR using gDNA templates (Fig. 3B & inset, Spearman coefficient *ρ* = −0.94; *p* = <0.0001). To determine whether variability in GDA copy number also exists within a single inhibition zone colony, we analysed the GDA copy number in single MinION nanopore raw reads from inhibition zone colonies, using 5 replicates. To display the highest GDA copy number possible in the length-limited nanopore raw reads, we shortened the GDA via. Wanner mutagenesis from 17 kbp to 7 kbp. As the nanopore sequencing generates sequence data from individual input DNA molecules without prior PCR amplification steps, gene copy number differences reflect differences present within a resistant colony^45,46^. Astonishingly, each colony revealed highly heterogeneous copy number distributions of GDAs, as shown for one colony (Fig. 3C). There were the entire GDA spanning raw reads from the same colony, displaying one but also five GDAs. The maximum GDA copy number detected was six. In another inhibition zone colony a maximum of 16 GDAs were detected and spanning reads with one GDA occurred (data not shown). This emphasises the enormous range of variation even within a single colony and demonstrates that genotypic plasticity extends to single-cell level.

Next, we asked how the observed GDA variability between and within colonies is related to growth under antibiotic CAZ pressure. To quantify colony appearance and growth arising from single-cells we used ScanLag to perform time-lapse imaging on agar plates^47^. We observed increased heterogeneity among resistant subpopulations in lag times of colony appearance, growing on antibiotic-containing plates (interquartile range (IQR) 693–984 min), whereas the ECC source population displayed homogenous lag times (IQR of 483–499 min.) in the absence of CAZ (Fig. 3D, Supplementary Fig. S6). qPCR analysis of genomic DNA of the colonies showed that early emerging colonies exhibited more GDAs than late emerging colonies in the presence of CAZ (Fig. 3E, F). To distinguish between subpopulations with long and short lag times, we chose a cut-off of 814 min., the median of the lag time of the resistant subpopulations. The average GDA copy number for colonies with short lag times was 21.49, whereas the average copy number for colonies with long lag times was 13.88 (Fig. 3E, unpaired students *t*-test *p* < 0.0001). Colonies harbouring high GDA numbers not only had shorter lag times (earlier onset of growth and therefore faster doubling), but also grew faster and accelerated growth more rapidly compared to colonies with lower GDA number (Fig. 3F). Surprisingly, despite their early onset of growth (short lag times), the growth rates of colonies growing without CAZ (green dashed lines in Fig. 3F) were relatively slow (lower slopes). In addition, they did not accelerate (curvature) as strongly as colonies with high GDA numbers that grew under CAZ exposure (Fig. 3F).

### Statistical modelling reveals a highly complex interplay between GDA, lag time and antibiotic treatment on growth of resistant sub-populations

Our observation that GDAs vary at the single cell level finally raised the question how lag time of colony appearance, colony growth and GDA number interact under CAZ exposure. To quantify these interactions, we fitted curvy-linear models that took GDA copy number, lag time and CAZ treatment as additive or interactive explanatory factors and colony growth of resistant subpopulations as response variable into account (Supplementary Table 7). The data we used were the ScanLag growth data described earlier (Fig. 3F). Comparing a first set of generalised linear models revealed that colony growth differed in their growth acceleration with time (comparing Model 1 that fixes growth acceleration to model 2 that allows for different acceleration of growth) (Supplementary Table 7). In addition, antibiotic treatment, lag time and amplification number not only simply added to explain variance among growth curves but all showed differences in growth acceleration. This implies that growth (slopes) and growth acceleration (curvature) differ with increased GDA copy number, that colony growth characteristics also differ between CAZ and CAZ free conditions and that growth rates and acceleration of growth differed among colonies with different lag times. All of these three effects, treatment, copy number or lag time, contributed to the differences in growth and growth acceleration and none of them was redundant. Combined, these statistical model comparisons highlight, by quantitative statistical support, the complex interplay between GDA, lag time and treatment (CAZ pressure), driving heterogeneity in growth of resistant subpopulations.

## Discussion

This study revealed HR to the third generation cephalosporine, CAZ in an *Enterobacter cloacae* complex (ECC) strain based on population plasticity originating from a GDA mechanism. Resistant sub-populations exhibited a high degree of resistance levels and a reversal of resistance after antibiotic withdrawal. We demonstrated that the molecular process of the heterogeneous phenotype is facilitated by the transient, unstable character of a plasmid-localised GDA of the AmpC-type β-lactamase *bla*_DHA-1_. The underlying genomic plasticity due to variable copy numbers of GDAs was detected at colony and even the single-cell level within purified bacterial colonies, and we examined the associated heterogeneity in fitness costs by exploring growth patterns and in competition assays.

The results highlight the problematic detection of HR which is complicated by the extent of heterogeneity among resistant subpopulations. Diverse bacterial strategies exist in response to antibiotic stress, e.g. mutations^48^, transcriptional regulation of stress related genes^49^, plasmid multiplication^50^ and GDA^51^. An additional phenotypic effect of *bla*_DHA-1_ due to increased copy number of the IncHI2/2A plasmid was ruled out since both resistant and susceptible ECC source population contained one plasmid copy per cell (Supplementary Fig. S7, Supplementary Table S5). Various molecular mechanisms enable cell-to-cell variations. Examples include stochastic variation of transcription factors and epigenetic changes or phase variation of certain gene loci^52^. For example, phase variation has similar mechanisms as GDA formation, but is mainly associated with an ‘ON or OFF’ state regarding the expression of a distinct phenotype^52,53^. The same applies for epigenetic switches concerning virulence of bacteria^54–56^. In conclusion, most of these mechanisms lack the fine-tuned evolutionary plasticity GDAs allow for.

The mechanisms underlying GDA are still a matter of debate. In addition to theories regarding unequal crossing over between homologous regions of sister chromosomes and the dependence or independence on recombination, the role of mobile elements and phenomena like strand slippage, tandem inversion duplication and single-strand annealing are discussed^43,44^. In many cases, GDAs are associated with direct repeated homologous areas flanking the GDAs^43,51,57,58^. We found for resistant subpopulations of ECC that the 17 kbp amplification of the plasmid was flanked by *qacE/sul1* (Fig. 2E)*. QacE/sul1* has been previously found in chromosomal GDA of *S*. Typhimurium^25,35^. In contrast, other authors have also demonstrated GDAs of varying lengths^59–61^ without considerable homologous regions. Those GDAs are discussed with initially random and RecA-independent mechanisms including ligation of DNA ends via gyrase activity^62^, strand slippage^63^, or pairing of single stranded regions^64^ and subsequent RecA-dependent amplification between homologous areas provided by the initial duplication^43^.

We confirmed GDA as the determining factor for CAZ HR in an ECC isolate using both genome sequencing (Illumina MiSeq and MinION technologies) and qPCR methods (Fig. 2B–F, Fig. 3A–C, E), and further showed that the underlying factor for growth heterogeneity is the varying copy number of GDAs (Fig. 3D–F). Previously, heterogeneity of resistant subpopulations has been shown in PAP assays^13^ by quantifying resistant subpopulations using increasing antibiotic concentrations, but these studies did not include growth data of subpopulations. In our study, using single colony ScanLag data, we were able to show that there is a wide phenotypic diversity regarding cell growth when the original population is exposed to CAZ, even at a single antibiotic concentration (Fig. 3D + F, Supplementary Fig S6). The distribution of GDAs not only varied between heteroresistant colonies, but also within single cells of a single, resistant colony based on nanopore sequencing data from single colonies (Fig. 3C). These findings align with previously shown cell-to-cell GDA-copy number heterogeneity of *lepB* upon arylomycin treatment in clonal populations of *E. coli* and *A. baumanii*^46^. The limiting factor to prove GDA copy number heterogeneity per raw read are the length of the raw reads. Only in both sides spanning reads, the number of GDA copies can be adequately detected. Remarkably, every single GDA dosage per cell translated into many different growth phenotypes in ECC resistant subpopulations with different scenarios on fitness. Fitness loss has previously been shown for GDA-driven HR^25,31^. Consistent with this, we also confirmed a fitness loss in competition assays for the GDA-containing resistant subpopulation in an antibiotic free environment (Supplementary Fig. S8). However, the fitness cost of resistance might not be high, as under experimental conditions comparing colony growth on antibiotic-free plates separately, the resistant phenotype and ECC source population showed no growth or lag time differences (Supplementary Fig. S9 and Supplementary Table S6). Within resistant subpopulations in a homogeneous antibiotic condition, bacteria with a higher number of GDAs and hence a greater level of resistance, outcompeted those with fewer GDAs (Fig. 3F). In the presence of antibiotics, therefore, increased GDAs do not appear to confer a fitness disadvantage. Further studies need to evaluate when GDA leads to a fitness disadvantage as described in the literature^65^.

Another hallmark of HR and GDA are their often reversible phenotype and transient genotype(s). In this study, we showed that not only the selection but also the reversal of GDA-driven resistant subpopulations is highly dynamic (Fig. 1D). Generally, the transience of resistant subpopulations has only been demonstrated in liquid culture, representing the average reversal at a populational level^25,31,35^. Here, we performed the resistance reversal experiments with single colonies originating from single bacteria on agar plates to examine loss of resistance at a subpopulation level.

As already shown for the closely related species *E. coli* and *Salmonella enterica*, respectively, the reversal of resistance is due to two factors: (1) The fitness burden of GDAs, which has been estimated to result in a fitness loss of 0.15% per DNA kilo base pairs in *E. coli*^65^ and (2), the inherent instability of GDAs due to deletional processes during the formation of GDAs^33^ which was estimated to have a mechanistic rate of 0.15/generation/cell in *Salmonella enterica*^66^. Thus, a successful reversal in antibiotic-free environments is dependent on the mechanism of GDA formation itself and the competition between those resistant cells with GDAs and less resistant cells which have lost GDAs. Such a competition may be higher in liquid media in which single cells are confronted with a large population of competing cells compared to assays examining individual, isolated cells after growth on plates where competition is limited to cells within one colony. Although no such specific values can be found for the genus *Enterobacter*, the shared affiliation to the family of *Enterobacteriaceae* and the close relationship to the genera *Escherichia* and *Salmonella* may explain the relatively long reversal time shown by some of the ECC resistant phenotype-replicates (Fig. 1D).

In our study we show, how antibiotic treatment, GDAs and lag times of single colonies influence the growth of resistant subpopulations through complex interactions (Supplementary Table S7). Such complexity challenges simple eradication of resistant subpopulation in clinics. Model comparison allowed us to evaluate the importance of the factors (lag time, treatment, GDA), with GDAs having the major impact on the heterogeneous, accelerated growth of resistant subpopulations (Supplementary Table S7). Consequently, GDA comprises a powerful strategy for ECC to survive antibiotic exposure. Within the host, additional variables may further influence resistant subpopulations’ growth behaviour. Nevertheless, our findings suggest treatment protocols against heteroresistant infections should focus on the minimisation of GDA triggering conditions in order to prevent accelerated growth of resistant subpopulations. In fact, initial results on this can be found in the literature: Gullberg and Pereira were able to show in their studies using *E. coli* and *S. enterica* as model organisms, that antibiotic concentrations below the so-called minimal selective condition (MSC) prevent the selection of GDA-bearing resistant subpopulations. However, it should be noted here that this strategy only works if the MSC is above the MIC of the source population. Otherwise, there would be no therapeutic effect. Finally, antibiotic combinations have been shown to successfully control heteroresistant infections. In a recent study, Band and colleagues impressively showed that antibiotics that had been shown to be harmful when used alone had additive effects when used in combination, leading to the complete eradication of pan-resistant *Klebsiella pneumoniae* strains. Such studies are of enormous importance, especially for clinical questions, as they can provide important information for a promising approach to the ever-worsening antibiotic resistance crisis^36^.

In summary, our studies on the gain and loss of GDAs demonstrate a GDA-driven population dynamics for a heteroresistant ECC strain and revealed traits involved in bacterial plasticity and fitness. In the absence of effective detection and treatment protocols for HR^13–15,35^, we believe our findings further emphasise the need for developing a heightened awareness of the phenomenon of HR among clinical isolates and further studies towards their efficient detection and treatment.

## Materials and methods

### Strains, media and antibiotics

The bacterial strain used in this study is a clinical ECC IMT49658-1 isolated from the wound of a horse in the equine clinic of the department of Veterinary Medicine at the Freie Universität Berlin (NCBI bioproject number: PRJNA1020684, biosample number: SAMN37527804, accession numbers CP135270-CP135274). Bacterial identification was performed with Vitek2 (GN-card) and via matrix-assisted laser desorption/ionisation-time of flight mass spectrometry (MALDI-TOF MS)-based identification with Bruker ultrafleXtreme in combination with flexControl (Version 3.4) and MBT Compass (Version 4.1) software (Bruker Daltonics, Billerica, MA, USA). In order to ensure clonality of the strain, a clearly isolated colony was the origin of the frozen stock. Additionally, species identification of the ECC IMT49658-1 isolate was done with WGS-data of this stock using ribosomal Multi Locus Sequence Typing (rMLST)^67^ and PubMLST (https://pubmlst.org/species-id), resulting in a 100% match for *Enterobacter hormaechei*.

IMT49658-1 defines the original ECC source population. In the presence of CAZ it exhibits a heteroresistant phenotype with susceptible and resistant population entities. The resistant phenotype IMT49658-3 represents these resistant subpopulations. For further investigations 9, additional inhibition zone colonies and 4 heat treated isolates were used of these strains (Supplementary Table S2). Cation-adjusted Mueller-Hinton II (MH II) agar and broth (Becton Dickinson and Company, Sparks, USA) was used for antibiotic resistance testing and LB broth (Lennox formulation; Carl Roth, Karlsruhe, Germany) for most other experiments. Unless noted otherwise, incubation of cultures was at 37 °C. CAZ was used from Sigma-Aldrich (St. Louis, USA). Heat treatment plasmid-curing was performed in brain-heart-infusion (BHI) media (Carl Roth, Karlsruhe, Germany), and CHROMagar^TM^ (Mast Diagnostica GmbH, Paris, France) plates. Columbia blood agar (5% sheep blood) (Becton Dickinson and Company, Sparks, USA) was used in reversal of resistance experiments.

### E-tests and agar-disk diffusion assays (antimicrobial susceptibility testing)

E-tests were performed with IMT49658-1 and IMT49658-3 with CAZ and CAZ-Avibactam combination (Biomérieux, Marcy-L‘Étoile, France). Agar-disk diffusion method was conducted with CAZ (Becton Dickinson BBL^TM^ SensiDiscTM, Sparks, USA) for IMT49658-1, IMT49658-3, IMT49658-1Δ*catA2*Δ*bla*DHA-1, IMT 49658-1Δc*atA2*Δ*recA*, IMT49658-1Δ*catA2*GDAshort and additionally with 12 colonies after heat treatment. For both antimicrobial susceptibility tests a bacterial density of McFarland standard 0.5, adjusted in NaCl, was incubated on MH II plates for 16–20 h at 35 °C according to CLSI VET01 (performance standards for Antimicrobial Disk and Dilution Susceptibility Tests for Bacteria isolated from Animals).

### Isolation of the resistant subpopulation

The suspension of a single inhibition zone colony from agar-disk diffusion assay to McFarland standard 0.5 in NaCl was streaked on a MH II plate and subsequently incubated with CAZ disk as described in the section (‘antimicrobial susceptibility testing’) above.

### Population analysis profile (PAP)-Assay

Exponential phase cultures (OD_600nm_ 0.5) of IMT49658-1, IMT49658-3 and a heat-cured isolate IMT49658-3_27x_P29 were set to a density of 10^8^ CFU/ml and subsequently diluted in 1 × PBS in 1:10 dilution steps until 10^3^ CFU/ml. 100 µl of appropriate diluted bacterial suspension were homogeneously applied to MH II plates with double increasing antibiotic concentration of CAZ, using triplicates for each concentration. Considering El Halfawy and Valvano´s suggestion^13^, we used a multiple concentrations of the breakpoint of CAZ, 16 µg/ml CAZ for *Enterobacterales*^68^ (Table 2A from ref. ^68^), resulting in 0, 2, 4, 8, 16, 32, 64, 128 and 256 µg/ml CAZ. Plates were incubated at 37 °C for 48 h and subsequently colonies were counted. After extrapolation of the chosen dilution the colonies were plotted in log_10_ CFU/ml on the y axis against the antibiotic concentrations on the x-axis. Assessment of HR was done by criteria of both, El Halfawy and Valvano^13^ and Band et al.^36^.

### Time-kill assays

Time-kill assays were conducted in 6 ml LB (Lennox) for IMT49658-1 and IMT49658-3 with 4-fold the MIC-concentration of CAZ for IMT49658-1, previously determined in E-tests. Note, that a MIC is not detectable in liquid broth-based MIC-assays for heteroresistant cultures, because the resistant subpopulation overgrows the initially major susceptible population. The inoculum preparation consisted of bacteria grown to exponential phase (OD_600nm_ of 0.5) adjusted to 10^7^ CFU/ml. After addition of the antibiotic at timepoint 0, bacteria were incubated at 37 °C and 200 rpm. Survival of bacterial cells were determined in triplicates in intervals of 2, 4, 6, 18 and 24 h after starting the experiment. To this end, aliquots were washed by centrifugation in order to remove antibiotic and the bacterial cell pellets were resuspended in 1 × PBS and diluted in 1:10 steps before plating. Cell counts were performed after 24 h incubation at 37 °C and values were plotted as percentage of the population at timepoint 0 (y-axis) against time (x-axis).

### Heat-treatment of IMT49658

Heat treatment aiming for plasmid curing was performed essentially as described by Schaufler et al.^42^ with some modifications. Briefly, heat treatment was performed with overnight cultures of IMT49658-1 and IMT49658-3 separately, incubating at 45 °C overnight in 5 ml BHI broth in a static, non-shaking manner. After every overnight incubation, 100 µl from the respective strain were transferred into fresh BHI broth and the incubation at 45 °C was repeated as described above. After every passage, a 1:10 or 1:100 diluted aliquot of ~10 µl was placed on a CHROMagar^TM^ plate and incubated at 37 °C overnight. Next, single colonies were picked and placed on a CHROMagar^TM^ plate with 25 µg/ml kanamycin and without kanamycin and incubated again overnight at 37 °C. The absence of kanamycin resistance indicates a possible heat disruption of the plasmid, because kanamycin-resistance-genes in IMT49658 are only localised on the plasmid and not on the chromosome. CHROMagar^TM^ plates were used because they detect contamination directly by colour of the CFU. CFU´s not growing on plates with kanamycin were double checked by re-incubating on plates with kanamycin. Confirmed non-growers were further investigated for plasmid disruption with PCR using primers for *bla*_DHA-1_ and *bla*_CTX_, both localised in the IncHI2/A plasmid.

### PCR for *bla*_DHA-1_ and *bla*_CTX_ in heat-treated CFU

PCR for genes of IncHI2/2A plasmid with colonies of heat-treated IMT49658 was performed with Promega GoTaq DNA Polymerase (M300) Protocol including the 5 × Green GoTaq® Reaction Buffer 1 (7.5 mM MgCl_2_). 1 µl of sample DNA was added to 49 µl Mastermix and 35 cycles of predenaturation (95 °C 180 s), denaturation (95 °C, 60 s), annealing (55 °C, 60 s), elongation (72 °C, 60 s) and endelongation (72 °C, 300 s) were performed.

### Reversal of resistance/stability analysis of antibiotic resistance

The resistant phenotype IMT49658-3 (resistant subpopulation) was streaked on columbia blood agar plate (with 5% sheep blood) and incubated over night at 35 °C (<18 h). A McFarland standard density of 0.5 was set in NaCl for each of ten individual colonies (replicates) from the blood plate and separately spread on MH II plates performing an agar-disk diffusion with CAZ as mentioned in the method section ‘antimicrobial susceptibility testing’. From the same McFarland suspensions, a single loop was streaked out on a blood agar plate for each replicate and incubated overnight at 35 °C together with the agar-disk diffusion plates. Next, after 16–20 h inhibition zones were measured and photographed. The reversal continued for each of the ten replicates by picking one single colony from blood agar, repeating the above-mentioned procedure over 21 subcultivations. Subcultivations were only conducted with colonies from blood agar without antibiotics, ensuring the chance for reversal. Daily agar-disk diffusion provided information for the current status of resistance of each replicate. An overview of the method provides Supplementary Fig. S1.

### Competition assays of resistant and heteroresistant phenotypes

Measurement of the competition assays between resistant (IMT49658-3) and ECC source population (IMT49658-1) were performed with both strains in one batch, competing for the same nutrients. A neomycin-cassette comprising plasmid pProbe´-gfp (LAA)^69^ (Addgene Cat. Nr. 40171) with mScarlet, a red fluorescence protein with a constitutive pnptII promoter, was used for distinguishing both phenotypes. Consequently, it was possible to distinguish both phenotypes by growth on neomycin and by colour of the colony (red). To constitute this plasmid-construct, we amplified mScarlet from plasmid pMRE135 and introduced it into pProbe´-gfp (LAA) between restriction sites NheI and EcoRV. In order to control for possible inference of the plasmid with growth, we used two competition assays at one time, one with pProbe´-gfp (LAA)+mScarlet in IMT49658-1 and a second one with the same plasmid in IMT49658-3. In parallel, two control assays were performed with plasmid containing either in IMT49658-1 or IMT49658-3 alone in order to detect plasmid loss over the course of the experiment in non-antibiotic containing media.

The competition assays were incubated at 37 °C, 200 rpm in 6 ml LB (Lennox) broth. Competition was measured by cell-count after 0, 8, 24 and 48 h. The starting inoculum for both strains were 200 µl of 2 × 10^6^ CFU/ml. After every time point, an aliquot of bacterial suspension was centrifuged and the remaining pellet was resuspended in PBS. Bacteria were plated at an appropriate dilution performed in 1 × PBS using a volume of 100 µl on plates (triplicates) with and without neomycin. Lastly, calculation of CFU on plates with and without neomycin after 24 h in 37 °C was used to calculate competitive index (CI) using the following formula: CI = (IMT49658-1 output/IMT49658-3 output)/(IMT49658-1 input/IMT49658-3 input).

### Growth analysis for resistant subpopulations with ScanLag

#### Experimental set-up

ScanLag experiments to determine lag times and growth rates of single colonies were performed as described by Levin-Reisman et al.^47,70^. To assess fitness loss between GDA carrying—and non-carrying isolates, experiments were conducted with IMT49658-1 and IMT49658-3 using MH II plates without CAZ. ScanLag experiments for resistant subpopulations were performed with IMT49658-1 on MH II plates with 32 µg/ml CAZ (2x breakpoint concentration of CAZ for *Enterobacterales*^68^ (Table 2A from ref. ^68^)). Here, only resistant subpopulations manage to grow and the majority of susceptible bacteria of IMT49658-1 do not grow.

Strains were grown from single colonies in 10 ml MH II broth at 37 °C and 200 rpm to exponential phase (OD_600nm_ 0.5) and diluted to 10^3^ CFU/ml before plating 100 µl of the bacterial suspension uniformly on MH II plates for experiments without CAZ. For experiments with CAZ, bacterial suspensions with 2.5 × 10^7^ CFU/ml were established and 100 µl of the suspension was uniformly plated on MH II plates with 32 µg/ml CAZ. Subsequently, plates were placed in plateholders of all scanners (Epson perfection V370 photo). For optimal contrast and absorption of moisture, sterile black felt cloth was placed between the plates and the plate lids as described in ref. ^47^. Incubation was performed at 33 °C due to the temperature resilience limitation of scanners. Scanners were controlled by the custom software UnixScanningManager (https://github.com/nirda/UnixScanningManager, commit 5d94cd3) on a Linux operating system (Ubuntu 20.04 LTS AMD 64). After an initial waiting time of 240 min, plates were scanned every 15 min. Incubation and image acquisition was done for 24 h.

#### Data analysis

Subsequently, images were analysed in Matlab R2020a (MATLAB. (2020).version 9.8.0.1396136 (R2020a). Natick, Massachusetts: The MathWorks Inc.) using a custom script incorporating previously published analysis image software NQBMatlab(https://github.com/oferfrid/NQBMatlab/tree/V16, commit 65306ce)^47^. Image analysis results were curated manually and, after removal of segmentation artifacts, exported to Microsoft Excel, providing information to appearance - and growth time^47,70^ per colony. Supplementary Fig. S6 was plotted with GraphPad Prism version 8.4.3 (686) for Windows, GraphPad Software, San Diego, California USA, www.graphpad.com.

The colony appearance time (lag time) is defined as the time at which a colony reaches the detection threshold area of 10 pixels. Growth times are defined as the time a colony needs to increase its area from 15 to 45 pixels (Supplementary Fig. S9 and Table S6). More detailed analyses of the growth data were done in programme R Core Team (2023). R: A language and environment for statistical computing. R Foundation for Statistical Computing, Vienna, Austria. URL https://www.R-project.org/. (Fig. 3C, Supplementary Table S7). To evaluate growth of single colonies, considering colony growth as change in area respectively pixel/time, (Fig. 3C, Supplementary Table S7), we right censored cells that merged during their growth and therefore used different definitions than the NQBMatlab definitions that exclude these cells^47^. Colonies that increased in pixel size of >1.5 times per 15 minutes and at the same time had at least 30 pixels were assumed to have merged and their growth was—after merging—right censored (i.e. data was excluded from merging onwards). Plotting of graphs (Fig. 3C) was done using R library ggplot2, Wickham H (2016). *ggplot2: Elegant Graphics for Data Analysis*. Springer-Verlag New York. ISBN 978-3-319-24277-4, https://ggplot2.tidyverse.org.

### Proteomics

Proteomic analysis was conducted with ECC source population (IMT49658-1) and resistant phenotype (IMT49658-3) each with 5 replicates. Initially, cell lysis with 20 mM ice-cold HEPES buffer was performed on pellets from exponential phase culture. A sonification step (UP100H; Hielscher Ultrasound Technology, Teltow, Germany) induced mechanical cell disruption, using a duty cycle of 1.0 and an amplitude of 100% for 45 s. In the next step in-solution trypsin digestion with 5 µg of total protein for each sample was carried out with the protocol of Wareth et al.^71,72^.

Nano LC-MS and data analysis was performed with derived peptides with an Ultimate 300 reversed-phase capillary nano liquid chromatography system attached to a mass spectrometer (Orbitrap Q Exactive HF, Thermo Scientific). Mass spectra´s raw data were analysed by MaxQuant software package version 1.6.14, including the Andromeda peptide search engine^73^. Default settings of MaxQuant were used except for label free quantification (LFQ) and match between runs (MBR). First, data were searched against the custom database for ECC with 5197 protein sequences generated from the whole genome sequence of IMT49658-1 (NCBI accession numbers CP135270-CP135274), a clinical ECC strain from our lab. Subsequently, protein-data filtering (contaminants, reverse hits and hits only identified by site) and statistical analysis was carried out with Perseus software version 1.6.14^74^. Proteins were considered if at least 3 of 5 replicates per group showed LFQ intensity values. Normal distribution imputation added missing values near the detection limit using the default settings (width 0.3, down shift 1.8). Using student’s *t*-test with a permutation-based FDR of 0.05 the differences of mean log_2_ fold protein LFQ intensity between the two experimental groups (IMT49658-3—IMT49658-1) were calculated. Mean log2 protein LFQ intensity differences with *p* < 0.05 and at least log2 fold change >+1 for IMT49658-3 and <−1 for IMT49658-1 were considered as proteins with altered expression. In perseus, a volcano graph with mean log_2_ fold protein LFQ intensity differences was plotted against −log_10_ *p*-values (Fig. 2A).

### DNA extraction

All DNA isolations were performed with standard protocols using the ‘Isolation of genomic DNA from bacterial suspension cultures’ protocol of Qiagen using the QIAamp DNA Mini Kit or the ‘Master pure genomic DNA purification Kit’ (Kit Epicentre) of Lucigen, sold by Biozym. The former was used with IMT49658-1 and IMT49658-3 for qPCR (GDA copy number detection) and WGS. Additionally, it was used with further inhibition zone and cell-lawn colonies from agar diffusion assays for qPCR and for Illumina Sequencing of 5 inhibition zone colonies and 4 heat treated samples of IMT49658-3. The standard protocol ‘Genomic DNA purification from gram negative bacteria’ from the Monarch HMW DNA Extraction Kit for tissue was used for five inhibition zone colonies of IMT49658-1Δ*catA2*GDAshort for nanopore sequencing with a subsequent DNA clean up and size selection step using High Prep^TM^ PCR magnetic beads (MagBio Genomics Inc.) in a bead to sample ratio 0.4 to preselect long DNA fragments for further long read sequencing (Oxford Nanopore MinION). When GDA copy number was of interest, cells were directly cultured from cryotube into LB broth omitting an interim culturing on LB plates in order to avoid GDA copy number loss by additional cell growth. For the same reason DNA extraction started as soon as the culture had an OD_600nm_ of 0.5.

### RNA extraction

RNA extraction was used for IMT49658-1 and IMT49658-3 for subsequent RT-qPCR gene expression measurements of β-lactamases using standard protocols of the Purification of total RNA from bacteria using RNeasy Mini Kit from Qiagen.

### Whole-genome sequencing (WGS) and genome analysis

#### WGS of IMT 49658-1 and IMT 49658-3, inhibition zone colonies IMT 49658-1 CFU1, 6, 9, 10 and 11

WGS was performed for both IMT49658-1 and IMT49658-3, as well as for inhibition zone colonies IMT49658-1 CFU1, 6, 9, 10 and 11. In brief, short-read sequencing was performed for all isolates on an Illumina NextSeq 550 platform using the NextSeq Reagent Kit v2.5 High Output (Illumina Inc., San Diego, CA, USA) and the Library Preparation Kit Nextera XT (Illumina), resulting in 150 bp paired-end reads and at an ~100× coverage. Additionally, samples were subjected to long-read sequencing using Oxford Nanopore MinION (Oxford, UK). MinION one-dimensional (1D) libraries were constructed using the SQK-RBK004 kit (Nanopore technologies, Oxford, UK) and loaded onto an R9.4 flow cell according to the manufacturer’s instructions. Sequencing data was then collected for a total of 48 h. Amounts of 1 ng and 400 ng of extracted DNA were used as starting materials for sequencing by Illumina MiSeq and MinION technologies, respectively. Quality control of raw sequencing data (quality filtering, adaptor removal) was performed using an in-house pipeline.

#### WGS of IMT 49658-1 ΔcatA2GDAshort CFU1_P1, CFU2_P1, CFU3_P2, CFU4_P2 and CFU5_P3

Isolates were subjected to long-read sequencing using Oxford Nanopore MinION (Oxford, UK). MinION one-dimensional (1D) libraries were constructed using the SQK-RBK114.24 kit (Nanopore technologies, Oxford, UK) and loaded onto an R10.4.1 flow cell according to the manufacturer’s instructions. Sequencing data was then collected for a total of 48 h. Amounts of 200 ng of extracted DNA was used as starting materials for sequencing by MinION technologies. Further, we performed adaptor trimming on sequencing data with porechop v0.2.4 (https://github.com/rrwick/Porechop) and a removal of reads <1000 bp with Filtlong v0.2.1 (https://github.com/rrwick/Filtlong). Finally, quality check for the length of raw reads was assessed with LongQC^75^ (https://github.com/yfukasawa/LongQC).

#### WGS of heat-treated isolates

The four isolates (IMT49658-3-25-45C-P9, IMT49658-3-26-45C-P9, IMT49658-3-26-45C-P23 and IMT49658-3-27-45C-P29) and IMT49658-1 ΔcatA2GDAshort CFU1_P1 were subjected to WGS with the Illumina MiSeq platform (Illumina, Inc., San Diego, USA). The 300 bp paired-end sequencing in 40-fold multiplexes was performed on the Illumina MiSeq platform with the MiSeq® Reagent Kit v3 (600 cycle) (Illumina Inc., San Diego, CA, USA), and the Nextera XT DNA Library Preparation Kit (Illumina) according to the manufacturer’s recommendations. The 300 bp paired-end reads were trimmed with Trim Galore (https://www.bioinformatics.babraham.ac.uk/projects/trim_galore/) and de novo assembled into contigs using Unicycler v0.4.9 at default settings.

#### Postprocessing of WGS data and genome analysis

Multiple approaches were utilised to reconstruct the genomes of the selected strains. Initially, closed genomes of IMT49658-1 and IMT49658-3 were generated through de-novo hybrid-assembly using a combination of both short- and long-reads with Unicycler v0.4.7^76^. The Unicycler algorithm first utilises the short-reads to reconstruct contigs which are then extended through the additional long-read sequences. Reconstructed genomes were then annotated using Bakta v1.6.1^77^ and RAST^78^, followed by SNP analysis with Snippy v4.6.0. In addition, hybrid-assemblies were generated for IMT49658-1 and IMT49658-3 using the assembly workflow of the CLC Genomics Workbench. This method accurately represents repetitive or amplified regions because it first utilises the long-reads to create a genomic scaffold and then uses the short-reads to improve the per-base-accuracy. GDA was identified within the assembly of IMT49658-3 through dot plot comparison with IMT49658-1 and verified by investigating the raw reads within the CLC Genomics Workbench. Additionally, a custom BLAST database was constructed to further assess the amount of GDAs present within IMT49658-3. Finally, closed genomes were reconstructed for IMT49658-1 CFU 1, 6, 9, 10, 11 and IMT49658-1Δ*catA2*GDAshort CFU 1_P1 using the long-read assembler Flye v2.9.1-b1780^79^, followed by additional quality-polishing using HyPo v1.0.3^80^ in conjunction with the Illumina short-reads.

A ring comparison of both IMT49658-1 and IMT49658-3 was created through the CGView platform (http://cgview.ca). Annotated genomes were hereby uploaded to the platform in order to generate a comparative ring visualisation including coding sequences and GC content (Fig. 2B).

In addition, genoms of IMT49658-1, IMT49658-3, of heat cured strains and of 5 inhibition zone colonies were analysed using Geneious software version 11.1.5 (https://www.geneious.com). Comparison of single genes or bigger genetic fragments were done with Geneious algorithm using the ‘mapping to reference’ and ‘pairwise alignment’ option. Heat cured strains missing plasmid located genes were additionally assessed with mauve alignment algorithm implemented in Geneious. Furthermore, we used the PlasmidFinder 2.1 of the Center for Genomic Epidemiology (CGE) of the technical university of Denmark version 2.0.1 (https://cge.food.dtu.dk/services/PlasmidFinder/) in IMT49658-1 and IMT49658-3. Resistance genes in IMT49658-1 and IMT49658-3 were detected with ABRicate (https://github.com/tseemann/abricate, commit 955d402) using the NCBI, MEGARES and ResFinder Databases and with Resistance Gene Identifier (RGI) (https://card.mcmaster.ca/analyze/rgi) using the Comprehensive Antibiotic Resistance Database (CARD).

### Gene expression analysis with RT-qPCR

RT-qPCR was performed with IMT49658-1 and IMT49658-3 for genes *AmpR*/*bla*_DHA-1_, *bla*_DHA-1_, *AmpR/bla*_ACT-16_, *bla*_TEM_ and *bla*_CTX_. Initially, we applied RNeasy Mini Kit (Qiagen) standard protocols to receive RNA, as indicated in the method-section ‘RNA-extraction’. Afterwards, we used RQ1 RNAse free DNAse (Promega) according to the manufacturer´s suggestion to erase remaining DNA. RNA concentration and purity was assessed with NanoDrop 1000 Spectrophotometer (Thermo Fisher Scientific) (A260/A280; ≥1.9, A260/A230 ≥ 1.9). Then a control-qPCR for RNA purity with 25 ng/well of DNAse digested RNA, using Power SYBR Green Master Mix (Thermo Fisher Scientific) was performed. In case no DNA contamination was detected (no fluorescence signals or a Ct bigger than 35), we generated complementary DNA (cDNA) according to the manufacturers instruction with 840 ng before approved RNA using RevertAID and Riboblock (reverse transcriptase and rnase inhibitor, Thermo Fisher Scientific). Finally, qPCR was conducted with 42 ng/well cDNA, performing technical triplicates per analysed gene, in 96 well plates (Eppendorf twin.tec® PCR Plate 96, unskirted, white). We used PCR cycler StepOnePlus^TM^ and Power SYBR Green Master Mix.

For gene expression analysis we used the 2^−ΔΔCt^-method. Here, normalisation was performed with the house keeping gene tryptophan synthetase β-chain (*trp*), resulting in ΔCt values. Final 2^−ΔΔCt^-values were generated after subtracting ΔCt values of IMT49658-3 (sample) from ΔCt values of IMT49658-1 (control), receiving ΔΔCt values and a relative fold change gene expression, using the formula 2^−ΔΔCt^. Finally, graphical presentation in GraphPad Prism was done showing Median and IQR per analysed gene.

### Plasmid copy number detection

For the detection of plasmid copy number comparing IMT49658-1 and IMT49658-3 representing resistant subpopulation with GDA on the plasmid we calculate the ratio of plasmid DNA and chromosomal DNA using the formula 2^−^^ΔΔCT^. Here, ΔCT is the difference in threshold cycles between two plasmid genes using primerpairs *repA*_1 & *repA_279* and the chromosomal genes *trpA*, *glnA* and *rpoB*. Relative fold change values of plasmid copy number in IMT49658-3 to IMT49658-1 was calculated from the subtraction of ΔCt values from resistant clones ‘sample’ to susceptible clones ‘control’ and the subsequent use of the 2^-ΔΔCt^ formula. For the detection of plasmid copy number per strain, IMT49658-1 and IMT49658-3 separately we used the formula 2^−ΔCt^, as previously described by Wang et al.^81^.

### Gene duplication-amplification (GDA) copy number inference using qPCR

GDA copy number was determined using qPCR from IMT49658-3, nine additional inhibition zone colonies and ten revertants of the reversal of resistance and early and late occurring colonies from ScanLag experiments with 32 µg/ml CAZ. In addition, GDA copy number was detected from IMT49658-1Δ*catA2*Δ*recA* after overnight growth in 16 µg/ml CAZ. Extraction of genomic DNA (gDNA) was performed as above for WGS and qPCR. We used 6,7 ng gDNA/well using the same 96 well plates, PCR cycler and Master Mix as described above for the RT-qPCR experiments. Ct values of *AmpR*/*bla*_DHA-1_ and *bla*_DHA-1_ represented the amplified region and the house-keeping gene *trp* the control sequence apart from the GDA. For GDA copy number calculation we used the 2^-ΔΔCt^-method. First, normalisation was conducted using *trp*, achieving ΔCt values and subsequently relative fold change values of GDA copy number was calculated from the subtraction of ΔCt values from resistant clones ‘sample’ from susceptible clones ‘control’ and the use of the 2^-ΔΔCt^ formula. Here IMT49658-1 or cell lawn colony outside the inhibition zone from the 9 mentioned inhibition zone colonies were used as control. To verify that the 17 kbp region was amplified, we used five different colonies from the inhibition zone of IMT49658-1 on CAZ and using primer pairs for the genes for dihydropteroate synthase, IS91 family transposase and the *sapA*, *pspA* and *qnrB* genes.

### Gene duplication-amplification (GDA) copy number inference using Illumina raw reads

GDA copy number was inferred with Illumina raw reads by calculating the following quotient: average coverage amplified product / average coverage non-amplified product (Supplementary File 1, Table 4). The amplified product of IMT49658 consists of ~17 kbp (51.5% GC content) for 4 of 5 inhibition zone colonies and ~28 kbp (54% GC content) for the remaining 1 inhibition zone colony. The non-amplified plasmid-product has 4 kbp (41.6% GC content). The average coverage of both products used in the aforementioned quotient were extracted from Geneious after mapping the Illumina raw reads to the respective reference sequence.

### Analysis of individual nanopore raw reads

In order to assess the heterogeneity of GDA copy numbers per inhibition zone colony (CFU), analysis of individual long reads spanning the amplified region was performed for five CFUs (IMT49658-1Δ*catA2*GDAshort CFU 1_P1, CFU2_P1, CFU3_P2, CFU4_P2, CFU5_P3). First, long ONT reads were mapped to the amplified region using minimap^82^ (v2.17-r941), in nanopore mode (‘-ax map-ont’), discarding secondary alignments (‘--secondary=no’) and only reporting hits in the resulting sam file (‘--sam-hit-only’). Reads containing the amplified region (e.g. mapped reads) were then extracted from the sam files using the ‘SamToFastq’ function of picard (v2.18.29-SNAPSHOT), http://broadinstitute.github.io/picard. Seqtk (v1.3-r106), https://github.com/lh3/seqtk, was then utilised to preprocess each read individually before re-mapping the previously reconstructed ‘upstream’ and ‘downstream’ regions of the GDAs back to the filtered read set using minimap. Specifically, ~20 kb surrounding the GDAs were extracted and defined as ‘upstream’ and ‘downstream’ regions. A custom Python script was then utilised to assess the presence, absence and GDA counts per mapped read. Finally, the average PHRED score was assessed per read. Results were merged and exported into tabular format using R (v4.4.0) for further assessment in Microsoft Excel. The final graph (Fig. 3C) was created for IMT49658-1 CFU 1_P1 with Inkscape vector graphics editor (Inkscape Project. (2020). *Inkscape*. Retrieved from https://inkscape.org) and Microsoft PowerPoint. All reads displayed had a PHRED score of at least 16 (3% error rate). After additional annotation through the Rast^78^ pipeline, GDA copy numbers in raw reads were once more manually verified within Geneious. Estimation of the lengths from both sides of spanning reads was conducted in Geneious counting 0.8 mm for 7 kbp.

### Wanner mutagenesis in *Enterobacter cloacae* complex (ECC)

Gene deletion mutagenesis of *catA2*, *bla*_DHA-1_, *recA* and the deletion of sequence for reduction of the plasmid p49658 GDA fragment from ~17 kbp to ~7 kbp was performed using the λ Red recombinase gene deletion/replacement protocol essentially as previously described^83^, with minor modifications. Briefly, a derivative of plasmid pKD4 was constructed in which the kanamycin-resistance cassette was replaced with a bleomycin/phleomycin-resistance cassette amplified by PCR using the plasmid pMSG360zeo^84^ (Addgene, cat. #27154) as a template, and cloned as an *Xba*I fragment into the *Xba*I sites of pKD4 to generate plasmid pKD4ble. Putative clones were sequenced to verify the sequence integrity and orientation of the FLP recognition target (FRT) and P1 and P2 priming sites flanking the bleomycin/phleomycin-resistance cassette. Plasmid pKD4ble was subsequently used as a template for generation of the mutagenic PCR products. For deletion of sequence to reduce the size of the GDA, plasmid p2795^85^ containing a kanamycin/neomycin-resistance cassette was used as a template for generation of mutagenic PCR products essentially as described above. After purification, the mutagenic PCR products were introduced by electroporation into electrocompetent *Enterobacter cloacae* (ECC) harbouring plasmid pSIM18^86^, encoding hygromycin-resistance and a heat-inducible, λ Red recombinase, as previously described^87^. Mutant selection was performed by plating of the bacteria to plates containing 15 µg/ml phleomycin. For sequence deletion of the GDA, selection was performed on plates containing 50 µg/ml neomycin. After verification of the gene/sequence deletions by PCR and sequencing, electrocompetent cells were prepared from the putative mutants and the antibiotic-resistance cassettes were eliminated by introduction of plasmid pCP20^88^ encoding Flp recombinase and subsequent screening for loss of the antibiotic resistance and additional PCR verification. Clones which had lost the antibiotic resistance were subsequently cured of plasmid pCP20 by growth at 37 °C, and screening for loss of plasmid-derived chloramphenicol resistance at 30 °C, as previously described^88^. To facilitate subsequent mutagenesis, deletion of the plasmid p49658-encoded, chloramphenicol-resistance *catA2* gene was performed first, and all further gene and sequence deletions were performed in the Δ*catA2* background in order to permit chloramphenicol selection and screening for plasmid pCP20. The primers used for all strain and plasmid constructions are listed in Supplementary Table S1.

### Statistics

Statistics was done using Graphpad Prism. Significance of the appearance times from ECC source population and its heteroresistant phenotypes in ScanLag was tested with Mann-Whitney-U Test (Fig. 3D, Supplementary Fig. S6). We did a spearman correlation to investigate the relation between GDA and inhibition zone of agar-disk diffusion assays (Fig. 3B, inset). Unpaired student *t*-test was performed on the average GDA-copy number of late and early occurring colonies in ScanLag (Fig. 3E). For deeper statistical analyses of colony growth in ScanLag (Fig. 3F, Supplementary Table S7) we used statistical model comparison based on AIC^89^, assuming that a ∆AIC > 2 suggest substantially better support of a given model. We fitted negative binomial distributed error structured generalised linear models with a log link function (package MASS function glm.nb). Model comparison included for all models the colony size (in pixels) as response variable, and as explanatory variables combinations of the factors time, a quadratic function of time, GDA, treatment (i.e. 32 μg/ml CAZ or control 0 μg/ml CAZ), and lag time. Interactions among these explanatory factors were explored with a focus on interactions among growth rates and GDA (time and amplification) (see Supplementary File 2, Table 7). To reduce model complexity (interactions) and for ease of interpretation, we set the time of all colonies when they passed the detection threshold (>10 pixel) to time = 0, even though lag times differed among colonies. The actual differences in when they reached the threshold is conserved in the factor lag time.

## Supplementary information

Supplementary Information

## Data Availability

Raw sequencing data, chromosomes and plasmids are deposited at NCBI: NCBI bioproject number: PRJNA102068. IMT 49658-1 can be found under the biosample number: SAMN37527804, Genbank accession numbers CP135270-CP135274. IMT 49658-3 can be found under the biosample number SAMN44249573, (Genbank accession numbers CP173198-CP173202). IMT49658-1Δ*catA2*GDAshort can be found under the biosample number SAMN44250108, and the raw reads in the sequence read archive SRX26352414. The mass spectrometry proteomics data have been deposited to the ProteomeXchange Consortium via the PRIDE^90^ partner repository with the dataset identifier PXD056728.
