# Supplementary material for: Heteroresistance in Enterobacter cloacae complex caused by variation in transient gene amplification events
Source: NPJ Antimicrob Resist. 2025 Feb 22;3:13. doi: 10.1038/s44259-025-00082-7 (PMC11846870; doi:10.1038/s44259-025-00082-7)
Supplement: Supplementary file 1 — Supplementary Information [file 44259_2025_82_MOESM1_ESM.docx]

Supplementary materials for

**Heteroresistance in *Enterobacter cloacae* complex caused by variation in transient gene amplification events**

J. Kupke*, J. Brombach, Y. Fang, S. A. Wolf, L. Thrukonda, F. Ghazisaeedi, B. Kuropka, D. Hanke, T. Semmler, N. Nordholt, F. Schreiber, K. Tedin, A. Lübke-Becker U.K. Steiner, M. Fulde*

*Corresponding authors: [Marcus.fulde@fu-berlin.de](mailto:Marcus.fulde@fu-berlin.de) (M.F.)

Published on….in ….doi….

Content of this supplementary file 1

-Supplementary Table 1. Primers used in this study

-Supplementary Table 2. Bacterial strains used in this study

-Supplementary Figure 1. Procedure of the reversal of resistance

-Supplementary Figure 2. List of proteins differentially expressed in resistant phenotype of *Enterobacter cloacae* complex (IMT 49658-3) relative to the *Enterobacter cloacae* complex (ECC) source population (IMT 49658-1)

-Supplementary Figure 3. E-test with ceftazidime (CAZ) and CAZ-avibactam for *Enterobacter cloacae* complex (ECC).

-Supplementary Figure 4. Elimination of heteroresistance in *Enterobacter cloacae* complex (ECC)

-Supplementary Table 3. Gene duplication-amplification (GDA) copy number measured with multiple genes from the amplified 17 kbp gene fragment

-Supplementary Figure 5. *Enterobacter cloacae* complex inhibition zone colonies

-Supplementary Table 4. Gene duplication-amplification (GDA) copy number results based on calculation from qPCR and coverage of Illumina raw reads (WGS)

-Supplementary Figure 6. Lag time distribution of *Enterobacter* *cloacae* complex (ECC) source population and its heteroresistant phenotype

-Supplementary Figure 7. & Supplementary Table 5. IncHI2/2A plasmid copy number in resistant (IMT49658-3) and ECC source population (IMT 49658-1) of *Enterobacter cloacae* complex

-Supplementary Figure 7. Lag time distribution of *Enterobacter* *cloacae* complex (ECC) source population and its heteroresistant phenotype

-Supplementary Figure 8. Competition assay for ECC source population vs. resistant phenotype

-Supplementary Figure 9 & Supplementary Table 6 Appearance time and growth time of ECC source population and resistant phenotype in ScanLag experiment without ceftazidime (CAZ).

-Supplementary Table 7. Competing models for colony growth curves

**Supplementary Table 1.** Primers used in this study

| Primer name | Detected gene | Sequence 5´- 3´ | Method |
| --- | --- | --- | --- |
| blaDHA-1 forward | *bla*_DHA-1_ | GTTCAGCCGTATAACCGTGCTTC | qPCR (gDNA, cDNA); PCR |
| blaDHA-2 reverse |  | TCACAATCGCCACCTGTTTTTCC |  |
| transreg_AmpR_forward | *ampR/bla*_DHA-1_ | CACACAGCCGAGTTGTTCTTCC | qPCR (gDNA, cDNA) |
| transreg_AmpR_reverse |  | CAGACGTTATCTCCCCCTTAACCC |  |
| bla_ACT-16_forward | *bla*_ACTH-16_ | AGCCCAGACCCTGATACATTGAC | qPCR (gDNA, cDNA) |
| bla_ACT_16_reverse |  | CAACGTGCAGGATATGGCGAAC |  |
| Regulator AmpR forward | *ampR/bla*_ACTH-16_ | TGACCCATTCAGCCATTAGCCAG | qPCR (gDNA, cDNA) |
| Regulator AmpR reverse |  | AAAGAATCATTCAGCACCGGCAG |  |
| blaTEM forward | *bla*_TEM-1_ | CTGCAACTTTATCCGCCTCCATCC | qPCR (gDNA, cDNA) |
| blaTEM reverse |  | ATACCAAACGACGAGCGTGACACC |  |
| Bla_CTX_M-3 forward | *bla*_CTX-M-3_ | TACCGAGCCGACGTTAAACACC | qPCR (gDNA, cDNA); PCR |
| Bla_CTX_M-3 reverse |  | TGTCGCCCAATGCTTTACCCAG |  |
| trpA (EC) primer forward | *tryptophan synthetase β-chain* | CTACGACAACGCATTTGCACAAC | qPCR (gDNA, cDNA) |
| trpA (EC) primer reverse |  | TCGATAATCTTCAGCGACTGCTCC |  |
| For_dihydro_qPCR | *sul1* | ATGCACCGTGTTTCAATCGACAGC | qPCR (gDNA) |
| Rev_dihydro_qPCR |  | CAATATCGGGATAGAGCGCAGGGT |  |
| For_transposase_qPCR | *IS 91 family like transposase (ISCR1)* | TTGGAGCGTGATGCCGAGAATACG | qPCR (gDNA) |
| Rev_transposase_qPCR |  | GCAAGGTTTGCAGGGTGAAGACTT |  |
| For_sapA_qPCR | *sapA* | ATCGTTACTGTCAGTTGCCCAACC | qPCR (gDNA) |
| Rev_sapA_qPCR |  | GCTCAGGTGGGTGTGAAAGTGAA |  |
| For_QnrB_qPCR | *QnrB*10 | TGTCCAGTTTGACGCCTTGCAAATC | qPCR (gDNA) |
| Rev_QnrB_qPCR |  | CGTTCAGTGGATCAGACCTCTCT |  |
| For_psp_qPCR | *pspA* | TCATCGGTGAACCCAGGAAACAACG | qPCR (gDNA) |
| Rev_psp_qPCR |  | CGTTACGCGAACGTCAAAGCGATAT |  |
| repA_For_1 | *repA* (1) | AACCTTGCATCGGTAGTGCTTGC | qPCR (gDNA) |
| repA_Rev_1 |  | CCAACCAGCTCAACAGTATCGCTT |  |
| repA_For_279 | *repA* (2) | TTGCTCGTGTTTTGAGGTTCAGACG | qPCR (gDNA) |
| repA_Rev_279 |  | CTGAAGGCAATTAAGGAGTTGGCCAA |  |
| glnA_qPCR_For | *glnA* | ACTTGTCACCAGAGAACAGGTTGG | qPCR (gDNA) |
| glnA_qPCR_Rev |  | CGCGACCTTCATGCCAAAACCAA |  |
| rpoB_qPCR_For | *rpoB*  mScarlet | TCACCTTACCTACCAGAATATCACCG | qPCR (gDNA)  constitution of pProbe´-gfp (LAA)+mScarlet |
| rpoB_qPCR_Fev |  | CCAGAAGAGATCACTGCCGACAT |  |
| For_konstit_mScarlet_NheI  Rev_konstit_mScarlet_EcoRV |  | GACTAGCTGCTAGCCCATAAACTGCCAGGAATTGGGGATC  ATCGGCATGATATCGGATTCTCACCAATAAAAAACGCCCG |  |
| p49658catA2H1P1   p49658catA2H2P2   49658catF3    49658catR2 | *catA2 pIncHI2/2A* | GCCTTCTGATTATTAATATTTTTCACTATTAATCAGAAGGAATAACCATGTGTAGGCTGGAGCTGCTTCGA  ATCCTGCCGGATACGGTGGCTTAAATACAGAATTAATTAATTTATTTCAGCATATGAATATCCTCCTTAG  GCGCCTGGTAAGCAGAGTT  CCAGACAGCTTACGGAGGAC | Wanner mutagenesis |
| p49658blaDHAH1P1  p49658blaDHAH2P2  p49658_blaDHA_Test_F  p49658_blaDHA_Test_R | *bla*_DHA-1_ | TGAATCTGACGATACTTGCCGCCGTTACTCACACACGGAAGGTTAATTCTGATGTGTAGGCTGGAGCTGCTTCGA  TACGGCCCCGGCGTATCCGCAGGGGCCTGTTCAGGAAAAAAATTATTCCAGCATATGAATATCCTCCTTAG  GCATGGGTGACATTCAGCTCAAT  AGCTGTCAGTGCCCGATACTC | Wanner mutagenesis |
| 49658_RECAH1P1  49658_RECAH2P2  49658_RECAF  49658_recAR2 | *recA* | AGTCCATGGTGAAGCGCAGTTGCTTCTCCCGGCATGACAGGAGTAATAATGTGTGTAGGCTGGAGCTGCTTCGA  CAGCAGCCCTTCATTTTTATCCGAGAGGATTAAAAGTCTTCGTTGGTTTCCATATGAATATCCTCCTTAG  CACTTGATACTGTATGACTATACA  ACGCACGGCAAGAATACG | Wanner mutagenesis |
| p49658_H1_GDAshort  p49658_H2mscarletGDAshort  For_mScarletGDAtest  Rev_mScarletGDAtest2 | Shortening of GDA fragment | TGCTGCGGCAAACTGATGTCAGTCTCAACACACAGAGGAAAATGTTGTCATTACCGTCGACCTCGAGGG  TTATACTTCCTATACCCAAGCCACCACAATAATCGCCAGCAACAGTGAAGG ATTACGCCAGCTGGCGAAA  TGGGCTGTTTTATGGAGGATG  GGCTGGACCGGCAGTTAAAATT | Wanner mutagenesis |

**Supplementary Table 2.** Bacterial strain used in this study: *Enterobacter cloacae* complex IMT 49658

| Strain details | Note | Phenotype to ceftazidime (CAZ) | WGS | Hints to genotype | Reference |
| --- | --- | --- | --- | --- | --- |
| IMT 49658-1 (reference for heteroresistant phenotype) | Source/parental population | Heteroresistant | Oxford Nanopore MinION & llumina | No gene duplication-amplification (GDA) | Our laboratory (NCBI biosample number: SAMN37527804, accession numbers CP135270-CP135274) |
| IMT 49658-3 (reference for resistant phenotype | Inhibition zone colony from IMT 49658-1* | Resistant | Oxford Nanopore MinION & Illumina | GDAs | This study (NCBI biosample number: SAMN44249573) |
| IMT 49658-1 CFU 1 | Inhibition zone colony from IMT 49658-1 * | Resistant | Oxford Nanopore MinION & Illumina | GDAs | This study |
| IMT 49658-1 CFU 2 | Inhibition zone colony from IMT 49658-1 * | Resistant |  | GDAs | This study |
| IMT 49658-1 CFU 3 | Inhibition zone colony from IMT 49658-1 * | Resistant |  | GDAs | This study |
| IMT 49658-1 CFU 4 | Colony material outside inhibition zone * | Heteroresistant |  | No GDAs | This study |
| IMT 49658-1 CFU 5 | Inhibition zone colony from IMT 49658-1 * | Resistant |  | GDAs | This study |
| IMT 49658-1 CFU 6 | Inhibition zone colony from IMT 49658-1 * | Resistant | Oxford Nanopore MinION & Illumina | GDAs | This study |
| IMT 49658-1 CFU 7 | Inhibition zone colony from IMT 49658-1 * | Resistant |  | GDAs | This study |
| IMT 49658-1 CFU 8 | Colony material outside inhibition zone * | Heteroresistant |  | No GDAs | This study |
| IMT 49658-1 CFU 9 | Inhibition zone colony from IMT 49658-1 * | Resistant | Oxford Nanopore MinION & Illumina | GDAs | This study |
| IMT 49658-1 CFU 10 | Inhibition zone colony from IMT 49658-1 * | Resistant | Oxford Nanopore MinION & Illumina | GDAs | This study |
| IMT 49658-1 CFU 11 | Inhibition zone colony from IMT 49658-1 * | Resistant | Oxford Nanopore MinION & Illumina | GDAs | This study |
| IMT 49658-1 CFU 12 | Colony material outside inhibition zone * | Heteroresistant |  | No GDAs | This study |
| IMT 49658-3_27_45C_P29 | Clone from heat-treated IMT 49658-3 | susceptible | Illumina | Two heat disrupted ares on IncHI2/2A plasmid -> *bla*_DHA-1_ negative | This study |
| IMT 49658-3_26_45C_P23 | Clone from heat-treated IMT 49658-3 | Susceptible | Illumina | Two heat disrupted ares on IncHI2/2A plasmid -> *bla*_DHA-1_ negative | This study |
| IMT 49658-3_26_45C_P9 | Clone from heat-treated IMT 49658-3 | Susceptible | Illumina | Two heat disrupted ares on IncHI2/2A plasmid -> *bla*_DHA-1_ negative | This study |
| IMT 49658-3_25_45C_P9 | Clone from heat-treated IMT 49658-3 | Susceptible | Illumina | Two heat disrupted ares on IncHI2/2A plasmid -> *bla*_DHA-1_ negative | This study |
| IMT 49658-3_24_45C_P11 | Clone from heat-treated IMT 49658-3 | Heteroresistant |  | *bla*_DHA-1_ positive | This study |
| IMT 49658-3_27_45C_P21 | Clone from heat-treated IMT 49658-3 | Heteroresistant |  | *bla*_DHA-1_ positive | This study |
| IMT 49658-3_28_45C_P4 | Clone from heat-treated IMT 49658-3 | Heteroresistant |  | *bla*_DHA-1_ positive | This study |
| IMT 49658-3_28_45C_P9 | Clone from heat-treated IMT 49658-3 | Heteroresistant |  | *bla*_DHA-1_ positive | This study |
| IMT 49658-1 (8) | CFU with short lag time ** | Resistant |  | GDAs | This study |
| IMT 49658-1 (9) | CFU with long lag time ** | Resistant |  | GDAs | This study |
| IMT 49658-1 (10) | CFU with long lag time ** | Resistant |  | GDAs | This study |
| IMT 49658-1 (11) | CFU from control plate without CAZ in ScanLag | Heteroresistant |  | No GDAs | This study |
| IMT 49658-1 (12) | CFU from control plate without CAZ in ScanLag | Heteroresistant |  | No GDAs | This study |
| IMT 49658-1 (13) | CFU with short lag time ** | Resistant |  | GDAs | This study |
| IMT 49658-1 (14) | CFU with short lag time ** | Resistant |  | GDAs | This study |
| IMT 49658-1 (15) | CFU with long lag time ** | Resistant |  | GDAs | This study |
| IMT 49658-1 (16) | CFU with long lag time ** | Resistant |  | GDAs | This study |
| IMT 49658-1 (17) | CFU from control plate without CAZ in ScanLag | Heteroresistant |  | No GDAs | This study |
| IMT 49658-1 (18) | CFU from control plate without CAZ in ScanLag | Heteroresistant |  | No GDAs | This study |
| IMT 49658-1 (19) | CFU with short lag time ** | Resistant |  | GDAs | This study |
| IMT 49658-1 (20) | CFU with short lag time ** | Resistant |  | GDAs | This study |
| IMT 49658-1 (21) | CFU with long lag time ** | Resistant |  | GDAs | This study |
| IMT 49658-1 (22) | CFU with long lag time ** | Resistant |  | GDAs | This study |
| IMT 49658-1 (23) | CFU from control plate without CAZ in ScanLag | Heteroresistant |  | No GDAs | This study |
| IMT 49658-1 (18) | CFU from control plate without CAZ in ScanLag | Heteroresistant |  | No GDAs | This study |
| IMT 49658-1 (pProbe´-gfp (LAA)+mScarlet) | Used in competition assay | Heteroresistant |  | No GDAs; Contains plasmid pProbe´-gfp (LAA) for neomycin resistance; with mScarlet red fluorescence protein |  |
| IMT 49658-3 (pProbe´-gfp (LAA)+mScarlet) | Used in competition assay | resistant |  | GDAs; Contains plasmid pProbe´-gfp (LAA) for neomycin resistance; with mScarlet red fluorescence protein | This study |
| IMT 49658-3 CFU 1.21 | Revertant replicate 1, 21^st^ subcultivation *** | Resistant -> heteroresistant |  | In process of losing GDAs | This study |
| IMT 49658-3 CFU 2.21 | Revertant replicate 2, 21^st^ subcultivation *** | Resistant -> heteroresistant |  | In process of losing GDAs | This study |
| IMT 49658-3 CFU 3.21 | Revertant replicate 3, 21^st^ subcultivation *** | Resistant -> heteroresistant |  | In process of losing GDAs | This study |
| IMT49658-3 CFU 4.21 | Revertant replicate 4, 21^st^ subcultivation *** | Resistant -> heteroresistant |  | In process of losing GDAs | This study |
| IMT 49658-3 CFU 5.10 | Revertant replicate 5, 10^th^ subcultivation *** | Resistant -> heteroresistant |  | In process of losing GDAs | This study |
| IMT 49658-3 CFU 6.17 | Revertant replicate 6, 17^th^ subcultivation *** | Resistant -> heteroresistant |  | In process of losing GDAs | This study |
| IMT 49658-3 CFU 7.13 | Revertant replicate 7, 13^th^ subcultivation *** | Resistant -> heteroresistant |  | In process of losing GDAs | This study |
| IMT 49658-3 CFU 8.19 | Revertant replicate 8, 19^th^ subcultivation *** | Resistant -> heteroresistant |  | In process of losing GDAs | This study |
| IMT 49658-3 CFU 9.13 | Revertant replicate 9, 13^th^ subcultivation *** | Resistant -> heteroresistant |  | In process of losing GDAs | This study |
| IMT 49658-3 CFU 10.21 | Revertant replicate 10, 21^st^ subcultivation *** | Resistant -> heteroresistant |  | In process of losing GDAs | This study |
| IMT 49658-1 (ECC) Δ*catA2*Δ *bla*DHA-1 | *Enterobacter* *cloacae* complex (ECC) knockout strain | Susceptible |  | Deletion of *catA2, bla*DHA-1 | This study |
| IMT 49658-1 (ECC) Δ*catA2*Δ*recA* | *Enterobacter* *cloacae* complex (ECC) knockout strain | Susceptible |  | Deletion of *catA2, recA* | This study |
| IMT 49658-1 (ECC) Δ*catA2* GDAshort | Deletion of ~ 7 kbp of the GDA. | Heteroresistant. Use of 5 inhibition zone colonies* CFU1_P1, CFU2_P1, CFU3_P2, CFU4_P2, CFU5_P3 | Oxford Nanopore MinION & (Illumina for IMT 49658-1 Δ*catA2* GDAshort source population) | Deletion of catA2,  7 kbp of the GDA: hypoth. gene, *YcjW*, *pspA*,*B*,*C*,*D*,*QnRB10* *sapA*,*B*,*C*, *O*, Oxidoreductase, *YdeJ*, putative membrane protein, hypoth. Gene, putative periplasmic protein | This study |

*in disk-diffusion assay with ceftazidime (CAZ) ** from 32 µg/ml CAZ plate in ScanLag-experiment
*** from reversal of resistance-experiment of IMT 49658-3 (resistant phenotype)

**Supplementary Figure 1**


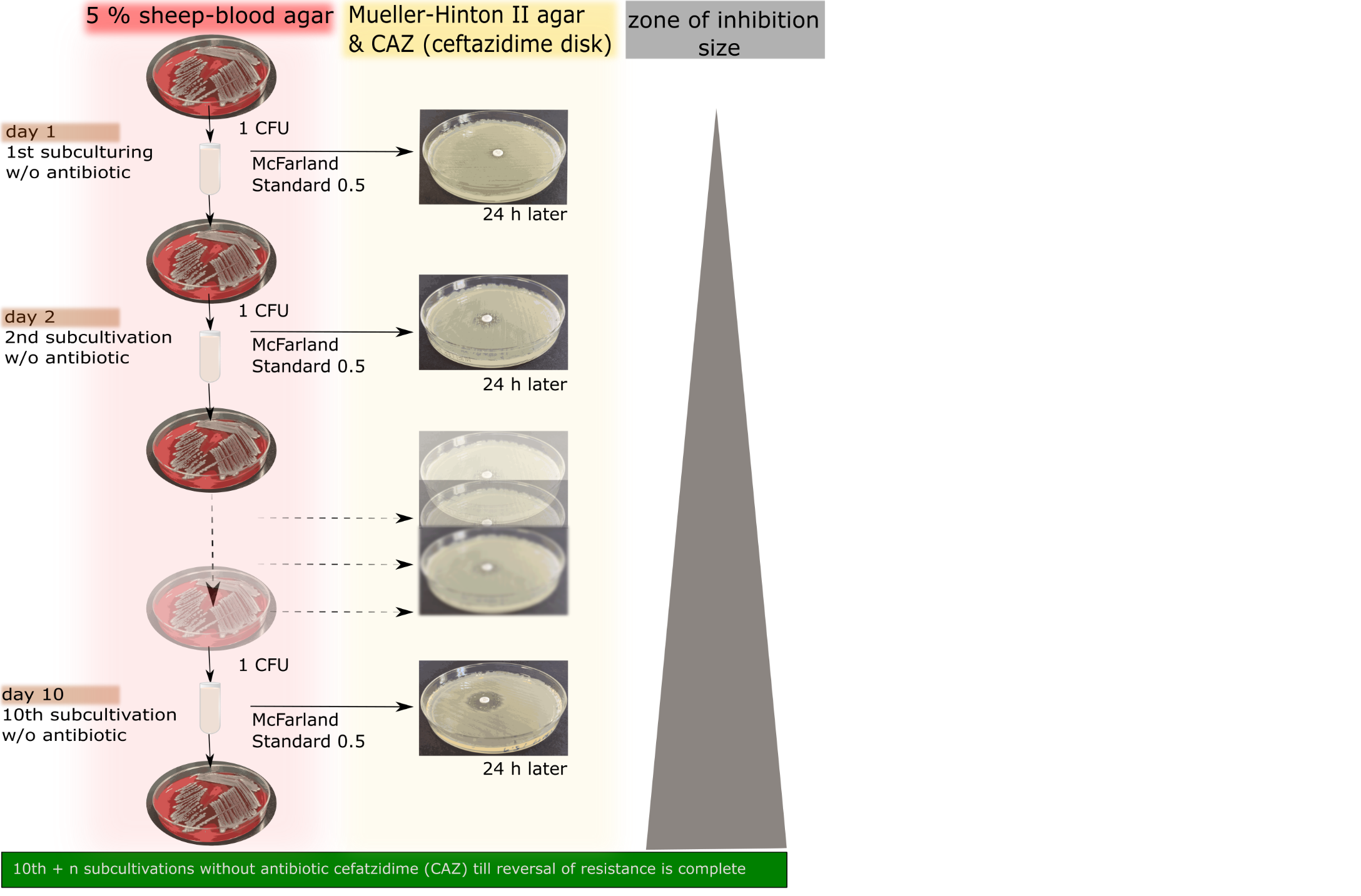


Supplementary Figure 1. Procedure of the reversal of resistance (in addition to the described method in the method section of the manuscript).

**Supplementary Figure 2**


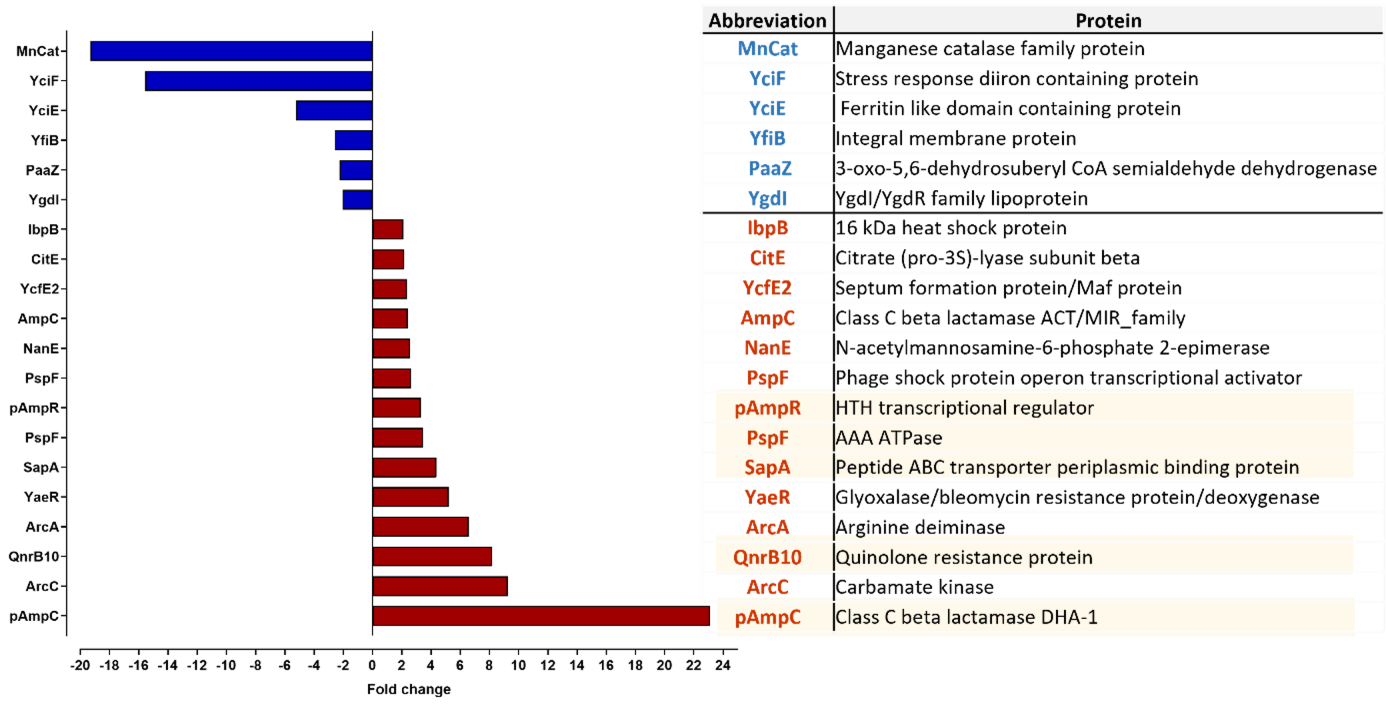


Supplementary Figure 2. List of proteins differentially expressed in resistant phenotype of *Enterobacter cloacae* complex (IMT 49658-3) relative to the *Enterobacter cloacae* complex (ECC) source population (IMT 49658-1). Proteins with at least 2-fold change in the expression level and values with –log _10_ *p*-value greater than 1.3 are considered as statistically significant. Downregulated proteins are shown in blue and upregulated proteins in red. Proteins from the gene duplication-amplification (GDA) which are upregulated in the ECC resistant phenotype are marked with yellow background.

**Supplementary Figure 3**

**
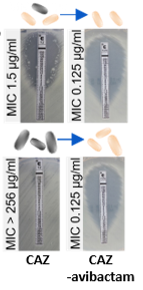
**

Supplementary Figure 3. E-test with ceftazidime (CAZ) and CAZ-avibactam for *Enterobacter cloacae* complex (ECC). In E-tests, ECC shows HR to CAZ and a MIC of ~ 1.5 µg/ml (upper left), while its resistant phenotype exceeds the maximum antibiotic concentration (> 256 µg/ml CAZ) of the E-test stripe (lower left). In the presence of CAZ-avibactam both phenotypes revert to susceptibility with a MIC ~ 0.125µg/ml (upper/lower right) and no inhibition zone colonies.

**Supplementary Figure 4.**


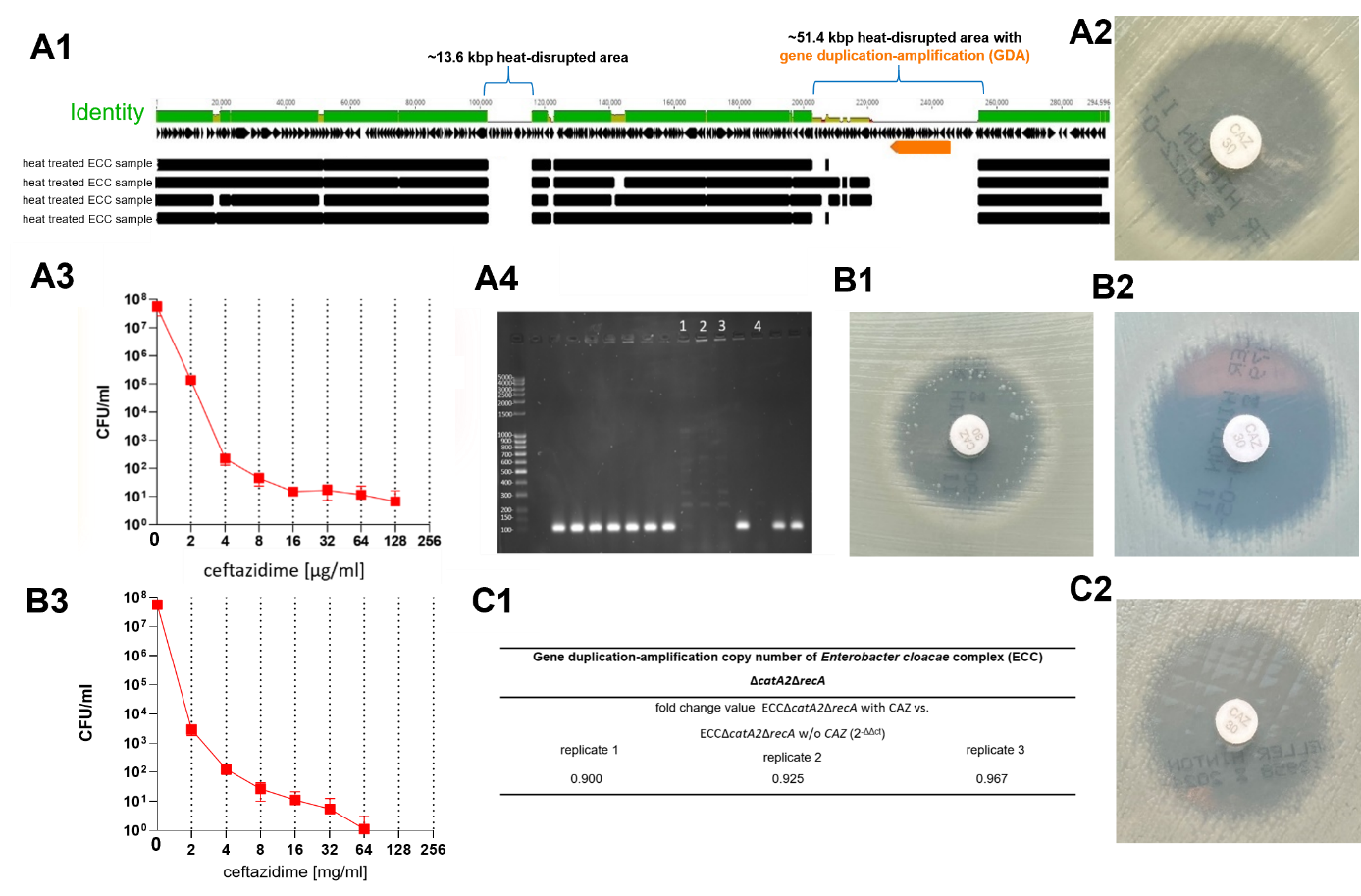


Supplementary Figure 4. Elimination of heteroresistance in *Enterobacter cloacae* complex (ECC). (A1) Heat treatment of ECC for plasmid curing. Mapping of Illumina reads from 4 heat treated samples of the resistant phenotype reveals two missing areas on the IncHI2/2A plasmid, one of them containing the GDA (orange arrow). Mapped contigs of the heat-treated samples are shown as longitudinal black bars. The extent of identity towards the reference is indicated in descending order of green (100%), brown (100-30%), and red (< 30%). (A2) Heat-treated samples lack heteroresistance due to the absence of inhibition zone colonies in agar-disk diffusion with CAZ. (A3) PAP assay of *_bla_*_DHA-1_ heat treated sample IMT 49658-3_27_45C_P29 shows a susceptible phenotype on rising concentrations of ceftazidime (CAZ), with 0.00003 % and 0.00002 % of cells from 0 µg/ml CAZ on plates with 2 and 4 fold the breakpoint concentration (32 and 64 µg/ml CAZ). Heteroresistance definition of Band et al. requires more than 0.0001 % of cells from 0 µg/ml CAZ on plates with 2 and 4 fold the breakpoint concentration (1). (A4) PCR detection of *bla*_DHA-1_ reveals 4 samples (labeled 1 to 4) *bla*_DHA-1_ negative. (B1) ECC Δ*catA2* shows inhibition zone colonies and inhibition zone as the ECC source population. (B2) ECC Δ*catA2*Δ*bla*_DHA-1_ has no inhibition zone colonies with the same inhibition zone (24 mm) as the ECC source population. (B3) ECC Δ*catA2*Δ*bla*_DHA-1_ shows 0.00000971 % and 0.00000195 % of the population size of 0 µg/ml CAZ at 2 and 4 fold the breakpoint concentration (32 and 64µg/ml CAZ), respectively. The above-mentioned cut-off for Heteroresistance is not reached. (C1) Three replicates of ECCΔ*catA2*Δ*recA* after overnight growth with 16 µg/ml CAZ show no GDA-copy number increase compared to growth without CAZ. (C2) ECCΔ*catA2*Δ*recA* lacks inhibition zone colonies in agar-disk diffusion.

**Supplementary Table 3.**

| **Fold change value (2^-ΔΔct^) of GDA copy number relative to no GDA containing ECC source population (IMT 49658-1)** | | | | | | | | |
| --- | --- | --- | --- | --- | --- | --- | --- | --- |
| **Genes from amplification-area** | | *sul1* | IS 91 family like transposase (ISCR1) | *sapA* | *QnrB10* | *pspA* | *bla*_DHA-1_ | *ampR/ bla*_DAH-1_ |
| **Inhibition zone colonies from heteroresistant phenotype (IMT 49658-1)** | CFU 1 | 12.57 | 6.93 | 35.83 | 36.05 | 33,84 | 29.48 | 28.75 |
|  | CFU 6 | 7.06 | 10.34 | 18.24 | 19.35 | 18.75 | 13.85 | 13.21 |
|  | CFU 9 | 6.26 | 8.41 | 18.18 | 19.35 | 15.98 | 13.20 | 13.07 |
|  | CFU 10 | 9.12 | 7.38 | 11.70 | 13.89 | 12.96 | 9.29 | 9.16 |
|  | CFU 11 | 6.22 | 9.36 | 17.67 | 17.93 | 16.56 | 15.94 | 16.31 |

Supplementary Table 3. Gene duplication-amplification (GDA) copy number measured with multiple genes from the amplified 17 kbp gene fragment. Here are listed the relative values of GDA copy number from five inhibition zone colonies, representing the resistant subpopulation, relative to the *Enterobacter cloacae* complex source population (IMT 49658-1). The mean value is derived from n = 3 technical replicates of each inhibition zone colony. Primers were selected for the genes from the entire 17 kbp gene fragment, and they all exhibit a greater gene copy number than the ECC source population. This demonstrates that the GDA has a stable length.

**Supplementary Figure 5**

**
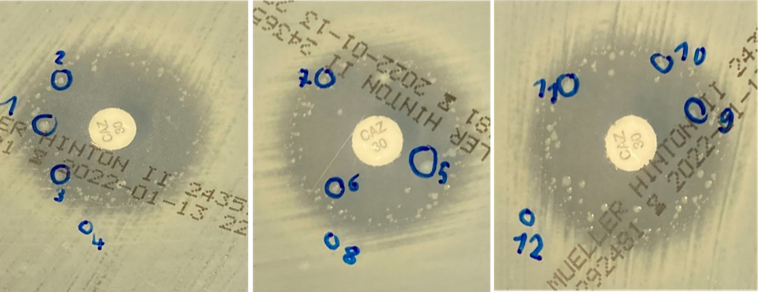
**

Supplementary Figure 5. *Enterobacter cloacae* complex inhibition zone colonies. 9 inhibition zone colonies (resistant subpopulations) from three different agar-disk diffusion assays with CAZ and cell lawn of each plate (CFU 4, 8, 12; photo) was used for the detection of gene duplication-amplification (GDA) copy number.

**Supplementary Table 4**

| **Inhibition zone colonies (resistant subpopulations) from *Enterobacter cloacae* complex** | **GDA copy number detected in qPCR** | **GDA copy number detected with Illumina raw read coverage calculations** |
| --- | --- | --- |
| IMT 49658-1 CFU 1 | 28.98 | 31.28 |
| IMT 49658-1 CFU 6 | 15.52 | 17.32 |
| IMT 49658-1 CFU 9 | 15.05 | 16.29 |
| IMT 49658-1 CFU 10 | 10.56 | 13.78 |
| IMT 49658-1 CFU 11 | 18.50 | 19.41 |

Supplementary Table 4. Gene duplication-amplification (GDA) copy number results based on calculation from qPCR and coverage of Illumina raw reads (WGS). qPCR results are displayed with the relative amount of GDA copy number compared to cell lawn outside the inhibition zone (CFU 4, 8, 12; photo) using 2^-ΔΔct^ values.

**Supplementary Figure 6**


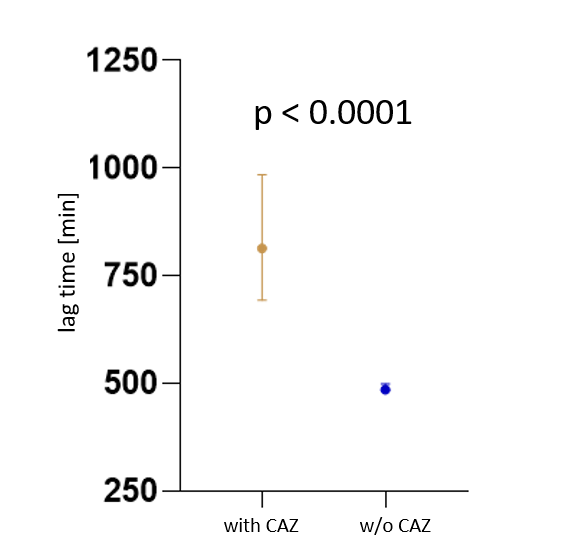


Supplementary Figure 6. Lag time distribution of *Enterobacter* *cloacae* complex (ECC) source population and its heteroresistant phenotype. ECC source population shows homogeneous lag times on plates without antibiotics in ScanLag (blue), but heterogeneous lag times (brown) when grown on plates with 32 µg/ml ceftazidime (CAZ), representing the resistant subpopulation. The dots indicate the median and the error bars the interquartile range (IQR).

**Supplementary Figure 7**


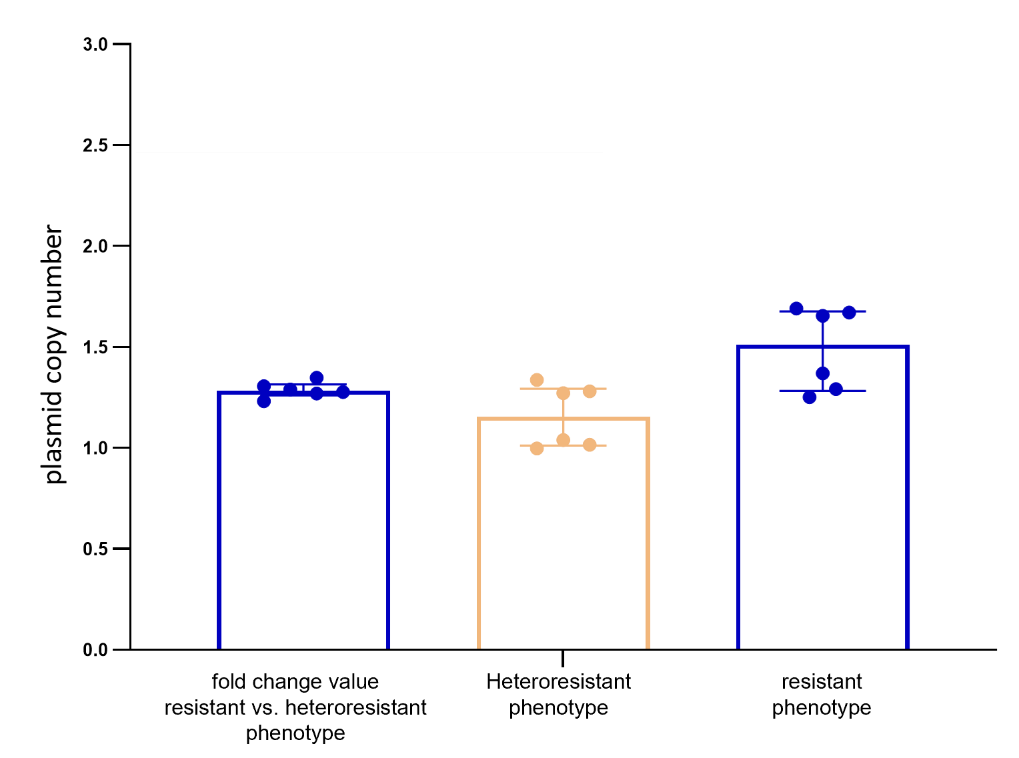


| **IncHI2/2A plasmid copy number in *Enterobacter cloacae* complex** | | |
| --- | --- | --- |
| fold change value resistant vs. ECC source population  (2^-ΔΔct^) | ECC source population  (2^-Δct^) | resistant phenotype  (2^-Δct^) |
| 1.27 | 1.16 | 1.49 |

**SupplementaryTable 5**

Supplementary Figure 7 & Supplementary Table 5. IncHI2/2A plasmid copy number in resistant (IMT 49658-3) and source population (IMT 49658-1) of *Enterobacter cloacae* complex. qPCR was performed with genomic (g)DNA. Three technical replicates of each primerpair was used. We assessed the Δct value from the substraction of plasmid genes – chromosomal house keeping genes. For the latter we used *trp*, *glnA* and *rpoB* and for the former we used two *repA* genes. Finally, with the formula 2^-Δct^ we calculate the plasmid copy number in the resistant phenotype and the ECC source population separately. Additionally, we calculated the relative fold change value of the plasmid copy number in the resistant phenotype compared to the ECC source population with the formula 2^-ΔΔct^. Both calculations show that the IncHI2/2A plasmid, containing the GDAs, is present with one copy in both phenotypes. Supplementary table 5 shows the median and supplementary figure 5 the median with IQR of qPCR results.

**Supplementary Figure 8**


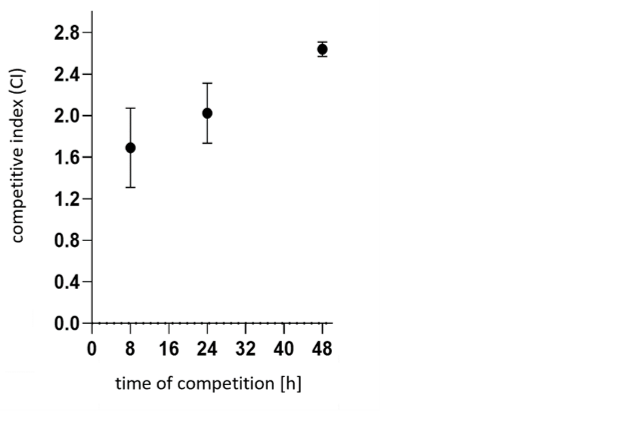


Supplementary Figure 8. Competition assay for ECC source population vs. resistant phenotype. Competitive indexes (CI) are shown after 8, 24, and 48 hours of competition in antibiotic-free media. Each datapoint shows mean and standard deviation of 2 replicate assays. The ECC source population outcompetes the resistant phenotype, as shown by the increasing CI over time. The CI is calculated with the following formula: CI = (ECC source population output / resistant phenotype output) / (ECC source population input / resistant phenotype input).

**Supplementary Figure 9.**


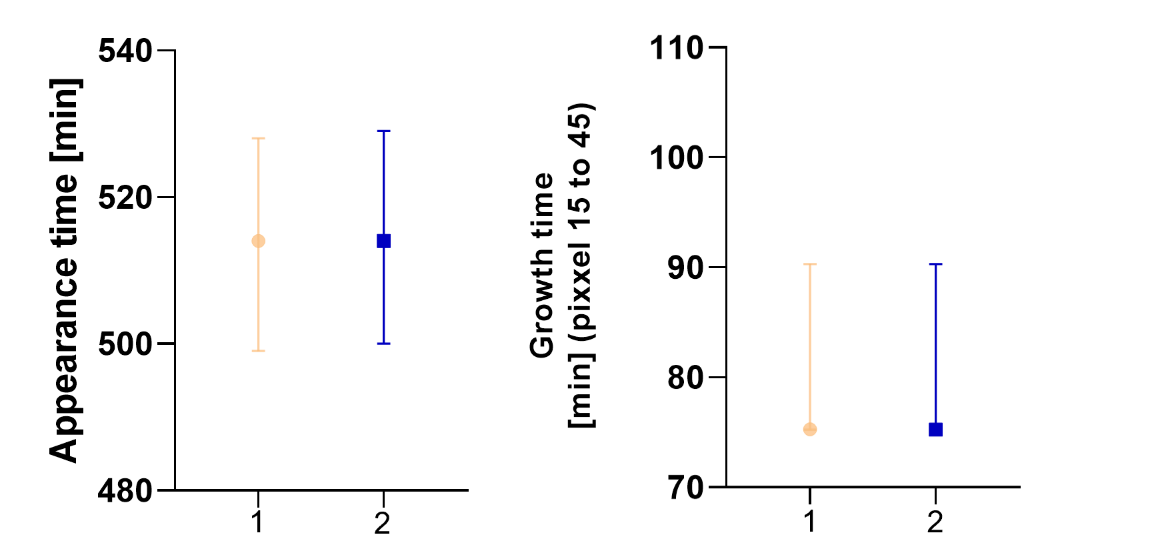


**Supplementary Table 6.**

| **Appearance time (lag time) [min]** | | | | **Growth time [min] (pixel 15-45)** | | |
| --- | --- | --- | --- | --- | --- | --- |
| **Strain/phenotype** | Number of colonies | Median [min] | IQR [min] | Number of colonies | Median [min] | IQR [min] |
| 1. IMT 49658-1 (ECC source population) | 1287 | 514 | 499-528 | 1234 | 75.27 | 75.25-90.3 |
| 1. IMT 49658-3 (resistant phenotype) | 1392 | 514 | 500-529 | 1371 | 75.27 | 75.25-90.3 |

Supplementary Figure 9 & Supplementary Table 6. Appearance time and growth time of ECC source population (beige) and resistant phenotype (blue) in ScanLag experiment without ceftazidime (CAZ). Both strains grow similar in ScanLag experiment on Mueller-Hinton II plates without CAZ. Median for lag time and growth time are the same, Interquartile range (IQR) is nearly identical for lag time and identical for growth time - the time in minutes a colony takes to grow from pixel 15 to 45 in the ScanLag system.

**Model comparison on growth related to amplification under ceftazidime (CAZ)**

**Supplementary Table 7** 1

| **Model Number** | **Model** | **DF** | **AIC** | **∆AIC** | **∆AIC** | **∆AIC** |
| --- | --- | --- | --- | --- | --- | --- |
| 1 | T+T^2+C+L+A | 7 | 4358 | 79 |  |  |
| 2 | T*T^2+C+L+A | 8 | 4279 | 0 | 65 |  |
| 3 | T*T^2+C+L | 7 | 4286 | 7 |  |  |
| 4 | T*T^2+C+A | 7 | 4429 | 150 |  |  |
| 5 | T*T^2+L+A | 7 | 4318 | 39 |  |  |
| 6 | T*T^2*C+L+A | 11 | 4244 |  | 30 |  |
| 7 | T*T^2*L+C+A | 11 | 4219 |  | 5 |  |
| 8 | T*T^2*A+C+L | 11 | 4214 |  | 0 | 121 |
| 9 | T*T^2*A+T*T^2*C+L | 14 | 4171 |  |  | 78 |
| 10 | T*T^2*A+T*T^2*L+C | 14 | 4114 |  |  | 21 |
| **11** | **T*T^2*A+T*T^2*L+ T*T^2*C** | **17** | **4093** |  |  | **0** |

Supplementary Table 7. Competing models for colony growth curves. Supplementary table 7 lists competing models for colony growth curves (colony size over time with curves being characterized by slopes and curvature) of colonies that differ in their amplification numbers and the prior exposure to CAZ (Treatment, i.e. resistant and source population, see also Fig. 3F of the main manuscript). We further evaluate lag times, the onset of growth defined as the time delay the initial cell needed to grow to a colony that passes the detection threshold of ScanLag of 10 pixel. The response variable is always colony size, the explanatory variables are time (T, the slope of the growth curves), the quadratic curvature or acceleration of growth with time (T^2), the amplification number (A), the lag time (L), and the treatment (C; control or 32 μg/ml). The latter is the only categorical variable. Additive factors in the model are indicated by a +; interactions among one or multiple factors are indicated by a *. Note, for these analyses we limited colony size to 500 pixels to account for decreasing pace in growth for larger colonies that heavily grow in three dimensions with the third dimension not being recorded by ScanLag. Lower AIC values provide better support for a given model and we consider a ∆AIC>2 as substantial better support of a given model. The best supported model is marked in bold font.. all interactions could be estimated for that we consider this model overparameterized.

The model comparisons analyse the data in Fig. 3 of the main text, though we limited size to a maximum of 500 pixels and assumed, for the statistical model fitting, that all colony growth started at the same time (i.e. all growth curves passed the x axis at time 0), the lag time parameter accounts for the delay in passing the threshold of 10 pixels.

Comparing model 1 (all growth curves have the same slopes and curvatures) to model 2 (growth curves differ in slopes and curvatures) shows that slopes and curvatures differ among growth curves, that is colonies differ in their exponential growth. Comparing model 2, 3, 4, and 5, shows that each factor (C, L, and A) contributes to explain the data as removing one of the factors results in a higher AIC. The factor that contributes the least in a simple additive only way is the number of amplification (∆AIC 7 when removing the factor, but see below for important interactions), followed by the treatment (∆AIC 39) and the lag time (∆AIC 150). This comparison might not be very informative, as for instance, lag time differences when only considered as additive term primarily highlights that the average size of colonies is smaller for late appearing colonies, which is not surprising, as those are right censored and therefore have not yet had the time to grow large. When considering the interaction between growth and one of the three other factors (C, L, or A) the results change (comparing model 2, 6, 7 and 8). Here, the amplification number is the most important predictor, implying that amplification explains important and strong differences in slopes and curvature among growth curves, with high amplification revealing fast growth (steeper slopes). The important part here might also be that the treatment (dashed growth curves vs. solid growth curves) explains less of the growth curve variation than the amplification.

There is additional complexity to only the growth and amplification, as models that include additional interactions between growth and lag time (model 10) or additional interactions between growth and treatment (model 9) are better supported than models that only have these factors as additive effects (model 8), with lag time interactions being more important than treatment interactions to explain growth rate patterns. The treatment interactions might be partly explained by the amplification number and growth interactions as they appear to be a little confounded (no CAZ exposure results in low amplification numbers). The most complex model (model 11) that allows for interactions between growth and each of the other factors (A, L, C) is the best supported model. With increasing complexity, biological interpretation becomes more and more challenging and higher parameterized models (not shown) do no longer allow to estimate all interactive terms, therefore we do not consider them here. They also indicate that we are approaching over parameterization and do not improve model fit in significant ways.

**Supplementary References**

1. Band VI, Hufnagel DA, Jaggavarapu S, Sherman EX, Wozniak JE, Satola SW, et al. Antibiotic combinations that exploit heteroresistance to multiple drugs effectively control infection. Nat Microbiol. 2019;4(10):1627–35.
